# Identifying Heteroatomic and Defective Sites in Carbon with Dual-Ion Adsorption Capability for High Energy and Power Zinc Ion Capacitor

**DOI:** 10.1007/s40820-021-00588-5

**Published:** 2021-01-21

**Authors:** Wenjie Fan, Jia Ding, Jingnan Ding, Yulong Zheng, Wanqing Song, Jiangfeng Lin, Caixia Xiao, Cheng Zhong, Huanlei Wang, Wenbin Hu

**Affiliations:** 1 Key Laboratory of Advanced Ceramics and Machining Technology (Ministry of Education), School of Materials Science and Engineering, Tianjin University, Tianjin, 300072 People’s Republic of China; 2 School of Materials Science and Engineering, Ocean University of China, Qingdao, 266100 People’s Republic of China

**Keywords:** Aqueous zinc ion capacitor, Dual-ion adsorption, Charge storage mechanism, High energy and power, Flexible and knittable devices

## Abstract

Aqueous zinc-based batteries (AZBs) attract tremendous attention due to the abundant and rechargeable zinc anode. Nonetheless, the requirement of high energy and power densities raises great challenge for the cathode development. Herein we construct an aqueous zinc ion capacitor possessing an unrivaled combination of high energy and power characteristics by employing a unique dual-ion adsorption mechanism in the cathode side. Through a templating/activating co-assisted carbonization procedure, a routine protein-rich biomass transforms into defect-rich carbon with immense surface area of 3657.5 m^2^ g^−1^ and electrochemically active heteroatom content of 8.0 at%. Comprehensive characterization and DFT calculations reveal that the obtained carbon cathode exhibits capacitive charge adsorptions toward both the cations and anions, which regularly occur at the specific sites of heteroatom moieties and lattice defects upon different depths of discharge/charge. The dual-ion adsorption mechanism endows the assembled cells with maximum capacity of 257 mAh g^−1^ and retention of 72 mAh g^−1^ at ultrahigh current density of 100 A g^−1^ (400 C), corresponding to the outstanding energy and power of 168 Wh kg^−1^ and 61,700 W kg^−1^. Furthermore, practical battery configurations of solid-state pouch and cable-type cells display excellent reliability in electrochemistry as flexible and knittable power sources.

**Supplementary Information:**

The online version of this article (10.1007/s40820-021-00588-5) contains supplementary material, which is available to authorized users.

## Highlights

- A unique dual-ion adsorption mechanism for zinc ion capacitor is enabled by a carbon cathode with defect-rich tissue, dense heteroatom dopant and immense surface area.
- The active sites on carbon surface for reversible dual-ion adsorption are identified by in-depth characterizations and DFT simulations.
- The zinc ion capacitor delivers unrivaled combination of high energy and power characteristics. The superb energy, power and cyclability are achieved in multiple cell configurations including coin cell and flexible solid-state pouch-/cable-type cells.

**Supplementary Information:**

The online version of this article (10.1007/s40820-021-00588-5) contains supplementary material, which is available to authorized users.

## Graphical abstract

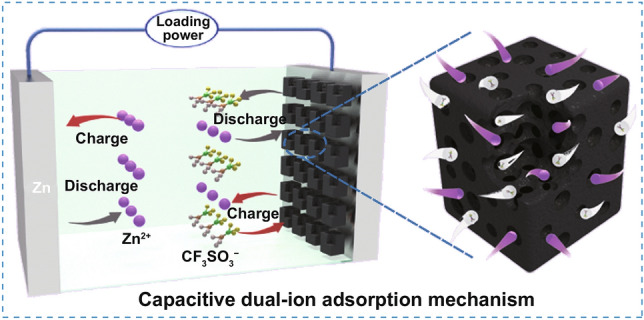

## Introduction

Owing to the high energy, high safety, low cost and potentiality of device flexibility, the aqueous zinc-based batteries (AZBs) become prospective candidates in the application scenarios of grid scale energy storage, electric vehicles and wearable flexible devices [1–4]. The advantages largely originate from the low redox potential, high gravimetric/volumetric capacity and moderate chemical activity of the zinc metal anode, as well as the non-flammable, low-cost aqueous electrolyte [5–8]. Based on the universal zinc anode and aqueous electrolyte, the diversity of the cathodes creates various derivative battery systems, including aqueous zinc-air battery [9–11], zinc-nickel battery [12, 13], zinc-manganese battery [14–17], zinc-vanadium battery [18–21], etc. The most essential difference among these well-defined battery systems lies in the charge storage mechanisms in the cathode side, which can be categorized into electrocatalysis [22–25], conversion reactions [26], intercalation reactions [27, 28], phase transformations [14, 29], etc. Regardless of the multifarious charge storage mechanisms employed in the cathodes, the ultimate objective being pursued is to obtain combined characteristics of high energy and power densities for the AZBs, which yet remains a significant challenge.

By introducing the capacitive charge storage mechanism in the cathode side of AZBs, the coupling of cathode and zinc anode assembles into a hybrid zinc ion capacitor, which is considered as a promising configuration to make the high energy and high power compatible [30–32]. Theoretically, the zinc anode can deliver a battery-level energy associated with the zinc plating/striping processes, while the facile kinetics of the capacitive cathodes guarantees a supercapacitor-mode power, which creates the possibility of combining the advantages of two stand-along technologies [33–36]. The practical implement of this strategy raises high requirements for the capacitive cathode material. First, the specific capacity of the cathode material should be as high as possible to match the zinc anode counterpart (820 mAh g^−1^), thus minimizing the total active mass. Therefore, the routine HelmHoltz double-layer capacitance is inadequate in this circumstance, but a synergy of various Faradic/non-Faradic charge storage mechanisms is highly desired. Second, the high power trait requires excellent capacity retention capability for the cathode at the extreme discharge/charge rates [37]; therefore, the sluggish kinetics of ion diffusion and charge transfer should be substantially eliminated.

Carbonaceous material is an important category of electrode material in various energy storage systems [38–42]. The wide applications are benefited from the high tenability in morphology, graphitic microstructure, porosity, surface chemistry, etc., for fulfilling different charge storage demands [43–46]. Carbon materials, for instance hollow carbon sphere [47], chemical activated graphene [30], heteroatoms doping porous carbon [48–50], etc., were attempted in hybrid zinc ion capacitors as the capacitive cathodes. These pioneering works are highly inspiring and reveal the great potential of applying carbons in advanced AZBs. Yet exploring carbons for high energy and power zinc ion capacitor is still in the infancy stage. According to our thorough literature survey, the current carbon cathodes-based zinc ion capacitors exhibited limited energy density of around 130 Wh kg^−1^. Also, the devices typically performed at moderate current densities of below 20 A g^−1^, leading to the power lower than 30 kW kg^−1^. More importantly, as aforementioned, the energy/power behaviors of AZBs correlate closely with the charge storage mechanism in the cathode. By far there is a shortage of systematical study on the charge storage mechanism of carbon cathode in zinc ion capacitors, which significantly obstructs the material design and device performance promotion.

In this work, we utilized a common biomass of protein-rich bone glue for carbon preparation. Via a templating/activating co-assisted carbonization procedure, the optimized carbon exhibits highly defect-rich graphitic tissue, 3657.5 m^2^ g^−1^ surface area, hierarchical porous structure and electrochemically active heteroatom doping of 8.0 at%. The zinc ion capacitor employing BGC cathode delivered maximum energy and power densities of 168 Wh kg^−1^ and 61,700 W kg^−1^, respectively. Both experiments and density function theory calculation illustrate that the superior electrochemical performance is essentially attributed to the unique dual-ion adsorption mechanism. The massive heteroatom moieties and lattice defects distributing on carbon surface provide large population of sites for both Zn^2+^ and CF_3_SO_3_^−^ adsorption, which exhibit capacitive kinetics in nature.

## Experimental Section

### Materials Synthesis

The bone glue biomass was purchased from Hunan Jusuo Biotechnology Co., Ltd. Sodium phytate and sodium hydroxide were received from Shanghai aladdin Biochemical Technology Co., Ltd. and Sinopharm Chemical Reagent Co., Ltd. The bone glue-derived carbons (BGCs) were prepared using bone glue as biomass precursor, sodium phytate as salt template and sodium hydroxide as activating agent. In a typical synthesis process, two solutions were firstly prepared by dissolving 2 g bone glue and 0.5 g sodium phytate in 20 and 5 mL deionized water at 60 °C, respectively. The solutions were mixed together and stirred at 60 °C for 4 h, and the obtained composite was loaded in an oven at 100 °C for 6 h to evaporate the residual water. The dried mixture was calcined at 450 °C for 1 h under argon atmosphere. The heating rate is 5 °C min^−1^. After grinding the pre-carbonized products with sodium hydroxide in a mortar in a mass ratio of 1: 2, carbonization of the mixture was processed under 650/750/850 °C for 2 h with a heating rate of 5 °C min^−1^ in argon. The obtained products were washed by HCl and deionized water for several times and dried at 80 °C for 12 h. The as-prepared bone glue-derived carbon was named as BGC-T, where T is the temperature in the second carbonization step. A commercial activated carbon (named AC) was utilized as received.

### Materials Characterization

To observe the morphology and microstructure of the carbons, a scanning electron microscope (SEM, Hitachi, S4800, 5 kV, Japan) and a transmission electron microscopy (TEM, JEOL, JSM-2100F, 200 kV, Japan) were employed. The X-ray diffraction (XRD) spectra were collected by Bruker D8 Advanced X-ray diffractometer (Bruker Corp., Billerica, MA, USA) with Cu Kα radiation. The gas absorption–desorption isotherm was characterized via autosorb iQ instrument (Quantachrome, US) at 77 K with nitrogen as adsorbate. The specific surface area of the carbons was estimated by Brunauer–Emmett–Teller (BET) method. The pore volume and pore size distribution were determined on the basis of density functional theory (DFT) model. Raman spectra were obtained using a Raman spectrometer (Lab RAM HR800, laser wavelength: 532 nm, laser power: 5 mW). X-ray photoelectron spectrometer (Axis Supra X, Japan) with Al Kα radiation (1486.6 eV) was used to collect X-ray photoelectron spectroscopy (XPS). For XPS ex situ electrodes preparation, sodium carboxymethyl cellulose (CMC) and alcohol solution were utilized as binder and solvent. After charging/discharging to the target voltage, the cell disassembly was completed within 30 s. The collected active materials were washed repeatedly with deionized water and vacuum-dried before XPS tests.

### DFT Calculation

The involved calculations were proceeded via the Vienna Ab initio Software Package (VASP 5.3.5) code based on the density functional theory (DFT), in which the Perdew–Burke–Ernzerhof (PBE) generalized gradient approximation and projected augmented wave (PAW) method were employed [51–53]. The plane wave cutoff energy was set to 400 eV in the all calculations. The Brillouin zone of the unit cell was sampled by Monkhorst–Pack (MP) method with Gamma centered in the 3 × 3 × 1 Monkhorst–Pack grid [54]. A k-point mesh was used for graphene structure optimizations, in which the convergence criterion for the electronic self-consistent iteration and force was set to 10^–5^ eV and 0.01 eV Å^−1^, respectively [55]. In this study, we constructed a 5 × 5 graphene surface supercell, including an atomic layer, to simulate the graphitic lattice. A vacuum layer of 12 Å was constructed to prevent the interaction between periodic layers. The ion adsorption energy (*E*_ads_) at various surface sites was estimated as follows:

1 $$ E_{{{\rm{ads}}}} = E_{{{\rm{total}}}} - E_{{{\rm{surface}}}} - E_{{{\rm{species}}}} $$

in which the *E*_total_, *E*_surface_ and *E*_species_ are representative of the total energy of the adsorbed species with graphene surface, the energy of the empty graphene surface and the energy of the specific adsorbed species in the gas phase, respectively.

## Results and Discussion

### Morphology and Physicochemical Characterization

**Scheme 1:**
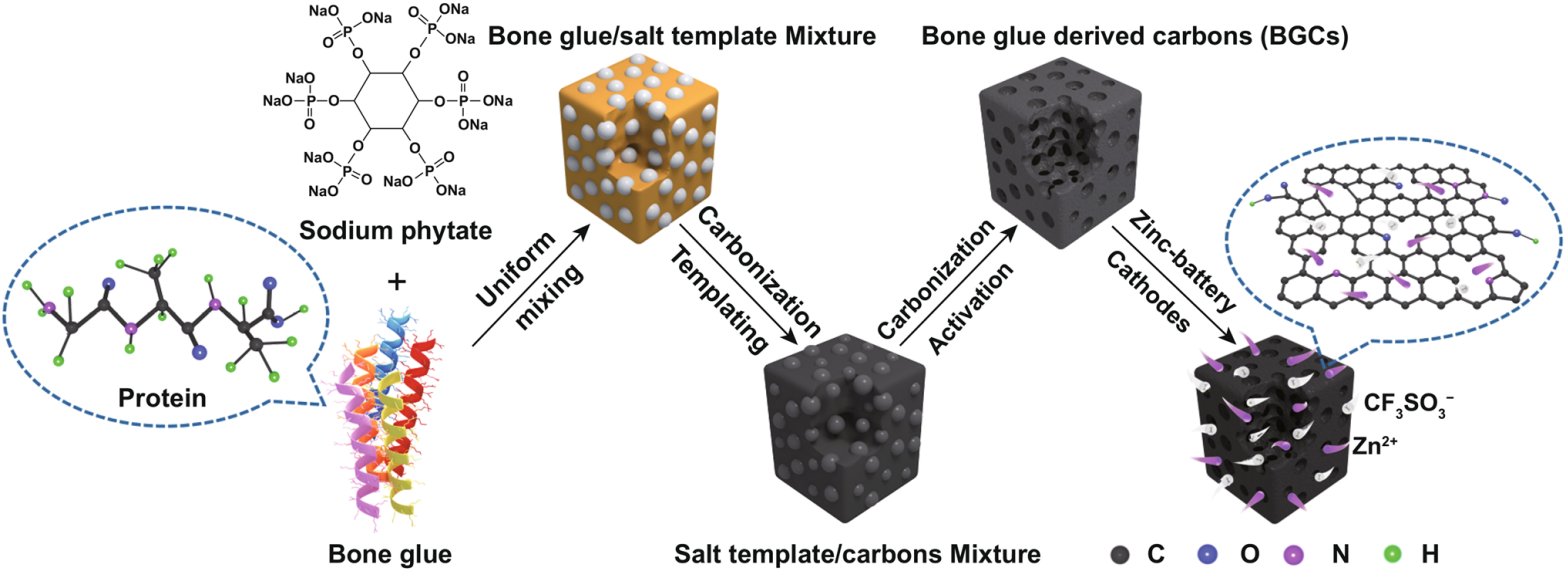
A schematic illustrating the preparation procedure of the bone glue-derived carbons (BGCs)

Scheme 1 demonstrates the transformation of the bone glue precursor into the carbonaceous materials (BGCs). Bone glue is a daily protein biomass mainly composed of polypeptide chains. The abundant elements of nitrogen and oxygen in the polypeptide can create intrinsic heteroatom doping for the obtained carbons. The preparation route involves two-step carbonization with the co-assistance of template and activation agents. It is of great importance that the bone glue melts above the temperature of 60 °C, when the gelatinous precursor can easily achieve uniform blend with the salt template of sodium phytate. The sodium phytate template not only generates macro-/meso-porosity after scarifying but also aids the sodium hydroxide to maximize the activation effect. According to the controlling experiments, the temperature in this templating/activating co-assistant carbonization procedure plays a vital role in tuning the physicochemical properties of the obtained carbon, as will be further discussed.

**Fig. 1:**
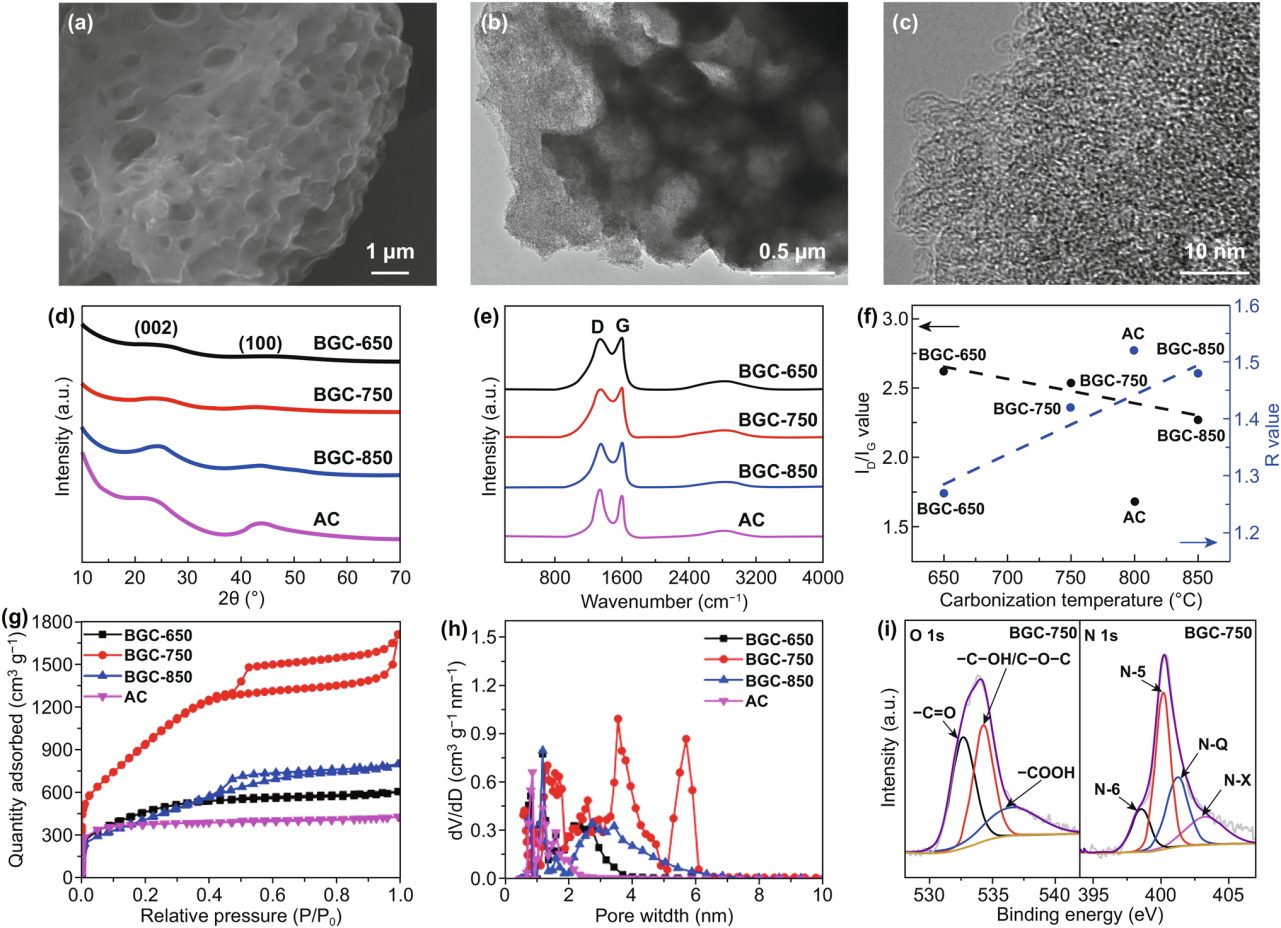
**a** SEM micrograph of BGC-750. **b** TEM micrograph of BGC-750. **c** High-resolution TEM micrograph of BGC-750. **d** XRD patterns of BGCs and AC. **e** Raman spectra of BGCs and AC. **f** *I*_D_/*I*_G_ and *R* values of BGCs and AC. **g** Nitrogen adsorption–desorption isotherms of BGCs and AC. **h** The corresponding pore size distributions. **i** O1*s* and N1*s* XPS spectra of BGC-750

Figure 1a shows the scanning electron microscopy (SEM) image of the optimized BGC sample with carbonization temperature of 750 °C (BGC-750), highlighting the honeycomb-like macroporous morphology. The SEM image with higher magnification (Fig. S1a) reveals the abundant 0.3–1 μm voids in the carbon bulk, which is largely attributed to the synergy of salt template removal and intense activating etch [43, 56]. As comparison, the BGC-650 shows visibly fewer macrovoids or cavities since the low temperature has much weaker activation effect (Fig. S1b). On the contrary, 850 °C is too high for activation; thus, the carbon framework collapsed and isolate macropores were largely destroyed [57], as evidenced by the mostly closed morphology of BGC-850 (Fig. S1c). The commercial activated carbon (AC) baseline exhibits solid micron-size particle morphology without any observable open macroporosity (Fig. S1d). Figure 1b demonstrates the transition electron microscopy (TEM) image of BGC-750. The interconnected macroporosity below 500 nm in the carbon bulk can be better observed. The high-resolution TEM image in Fig. 1c displays a highly disordered graphitic microstructure of BGC-750. Negligible continuous graphene fringe appears. Instead, massive micropores and defects clearly present on the surface.

The graphitic structure of BGCs and AC was first studied by XRD. Figure 1d exhibits the resulted spectra. The BGCs and AC all demonstrate broad (002) and (100) humps that are resulted from the amorphous carbonaceous tissue. In order to distinguish the delicate difference in graphitic microstructure among the samples, we utilized an empirical parameter *R* as an indicator of the number of carbon sheets arranged as single layers [58]. The method for *R* calculation and the corresponding values are shown in Fig. S2 and Table S1. Basically a lower *R* value is reflective of more randomly stacked graphene layers without ordered orientations, which closely correlates with the degree of disorder. Referring to Fig. 1f, the *R* values of BGCs almost linearly increase upon higher carbonization temperature. Nonetheless, AC has an exceptionally high *R* value. This is the first evidence of the more disordered graphitic microstructure of BGCs as comparing to AC. Figure 1e shows the Raman spectra of BGCs and AC. All spectra have characteristic D and G bands, which, respectively, represents the defective graphitic microstructure and the *sp*^2^-hybridized graphitic domains [46, 59]. Therefore, the degree of graphitic disorder and the defect concentration can be quantitatively evaluated by the intensity ratio of the two bands (i.e., *I*_D_/*I*_G_) [60]. As demonstrated in Fig. S3, the *I*_D_/*I*_G_ values were calculated based on the integral areas under curves. The large *I*_D_/*I*_G_ values of BGCs (Table S1) indicated the high degree of disorder and large population of defects in their graphitic lattices [43, 60, 61]. As shown in Fig. 1f, the *I*_D_/*I*_G_ also fits into a linear relationship with carbonization temperature. AC has a distinctly lower *I*_D_/*I*_G_ value that largely deviated from the BGC line. Both *R* and *I*_D_/*I*_G_ values reveal that BGCs have larger degree of disorder containing much higher population of defects than the routine active carbons. We claim that the unique synthesis procedure aforementioned for BGC plays crucial role in creating such a special carbon tissue.

To characterize the porous structures of BGCs and AC, nitrogen adsorption/desorption isotherms analysis was performed. In Fig. 1g, the BGCs and AC present a typical type IV and I adsorption/desorption isotherms, respectively. For BGCs, the sharp rise of gas adsorption at relatively low pressure range (*P*/*P*_0_ < 0.01) and the continuous increase at medium pressure range (0.1 < *P*/*P*_0_ < 0.5) are due to the existence of the micropores. The distinct hysteresis loop at the pressure range of 0.5–0.95 is reflective of mesoporosity [49, 62]. Specific to BGC-750, a slight rise at the high pressure range (0.95 < *P*/*P*_0_ < 0.99) can be observed, which proves the existence of the macropores [63], whereas for AC, the platform occupying the whole pressure region of the isotherm indicates that the porosity is mainly composed of micropores [64]. These observations are in line with the SEM and TEM results. As shown in Table S1, the temperature significantly alters the Brunauer–Emmett–Teller (BET) surface areas and pore volumes of BGCs. At lower temperature of 650 °C, the activation effect is too weak to generate massive pores, leading to the low surface area and pore volume for BGC-650, whereas the high temperature of 850 °C accelerates the graphitization which can seal the micropores and small mesopores [57, 65]. The optimized temperature of 750 °C maximizes the templating/activating effect and hence leads into the highest surface area and pore volume of 3657.5 and 2.428 cm^3^ g^−1^, 2.9 and 4.5 times the values of commercial AC. Figure 1h displays the pore size distribution of BGCs and AC. AC is almost mesopore free, while BGCs possess both micro- and meso-porosities. BGC-750 has distinct mesoporosity with diameters of 2.5, 3.5 and 5.7 nm. Combining with the micro-/meso-pores and macro-size voids, BGC-750 exhibits a favorable hierarchical porous architecture.

The surface chemistries of the carbons were investigated by X-ray photoelectron spectroscopy (XPS). As expected, the protein-rich precursor results into heteroatom-doped BGCs (Fig. S4a and Table S1). BGC-750 has moderate contents of both nitrogen (2.28 at%) and oxygen (5.72 at%) among BGCs; nonetheless, the highest surface area significantly magnifies the surficial heteroatom moieties exposed to electrolyte. The heteroatom information is also revealed in the high-resolution C 1*s* spectra. Per Fig. S4b-e, the components of C=C/C–C, C–O/C–N, C=O, and COOH could be clearly discriminated [64, 66]. The deconvolution of BGC-750 O 1*s* and N 1*s* spectra is shown in Fig. 1i. The three peaks in O 1*s* represent quinone-type groups (C=O, O-I), phenol groups/ether groups (C–OH/C–O–C, O-II) and carboxylic groups (COOH, O-III) [67]. For N 1*s*, the four peaks present pyridinic-N (N-6), pyrrolic-N (N-5), quaternary-N (N-Q) and oxidized N (N-X), respectively [63, 67, 68]. Other BGCs exhibit identical functional group configurations (Fig. S5), but there are diversities in the relative ratios of different oxygen and nitrogen species as a result of temperature change (Table S2). For BGC-750, O-I and N-5 occupy the largest proportions, which are 40.05% and 40.81% of all oxygen and nitrogen groups. For comparison, commercial AC is oxygen containing but totally nitrogen free.

### Electrochemical Performance of BGC- and AC-Based Zinc Ion Capacitors

**Fig. 2:**
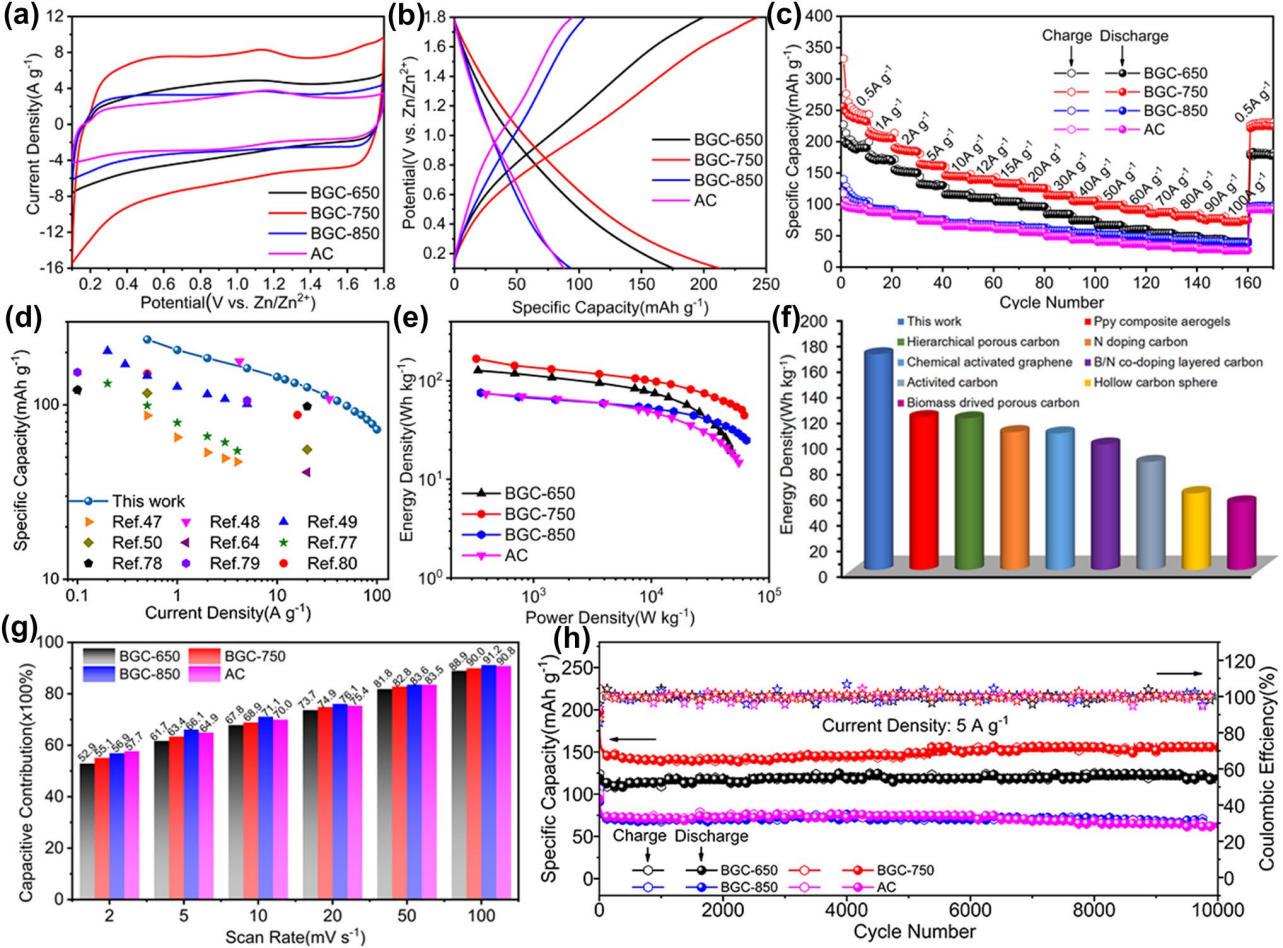
Electrochemical performance of BGCs and AC cathodes-based zinc ion capacitors. **a** CV curves at 20 mV s^−1^. **b** Galvanostatic charge–discharge profiles at 1 A g^−1^. **c** Specific capacities at various current densities. **d** Rate performance comparison of BGC-750-based zinc ion capacitors versus literature reported values. **e** Ragone plot of BGCs and AC-based zinc ion capacitors. **f** Maximum energy density of BGCs versus the state-of-the-art carbon cathodes. **g** Normalized contribution ratio of capacitive capacities at different scan rates. **h** Cyclabilities of BGCs and AC at 5 A g^−1^

We constructed aqueous zinc ion capacitors by coupling the BGCs, AC as cathodes and zinc foils as anodes. Zn(CF_3_SO_3_)_2_ aqueous solution (3 M) was utilized as the electrolyte, which ensures high Zn plating/stripping round-trip efficiency in the system [30]. Figure 2a shows the cycling voltammetry (CV) curves of BGCs and AC-based zinc ion capacitors at 20 mV s^−1^. All the curves exhibit near-rectangular shapes in the voltage window of 0.1–1.8 V with negligible evidence of oxygen or hydrogen evolution side reactions. Some sub-structures in the CV curve, for instance, the small cathodic humps at 1.0/1.2 V, are reflective of certain redox processes involved [69]. As shown in Fig. S6, the near-rectangular shape of CV curve maintained well for BGC-750 as the scan rate increased to 500 mV s^−1^, while severe distortions appeared for other BGCs and AC. This phenomenon is a clear evidence of the more facile ion transport kinetics in BGC-750 than that of in others, which is largely benefited from the wide-open hierarchical porosity [62, 64, 70]. The galvanostatic charge–discharge (GCD) tests were conducted at current densities from 0.5 to 100 A g^−1^. Figure 2b exhibits the obtained GCD profiles at 1 A g^−1^. The symmetrical quasi-triangular shape of the profiles is in line with the CV curves and also demonstrates the outstanding Coulombic efficiency (CE) [69]. The IR drop reflects the resistance of charge transfer in the electrode. As shown in Fig. S7 and Table S3, the IR drop values of BGC-750 are comparable to that of the highest graphitized BGC-850, distinctly lower than BGC-650 and AC. In addition, the IR drop of all the samples increased at higher current density resulted from the higher charge transfer resistance.

Figure 2c includes the gravimetric capacities of BGCs and AC cathodes at various current densities. The GCD profiles for first three cycles of these electrodes are shown in Fig. S8. In these initial cycles, the charge capacities are typically larger than discharge capacities. This phenomenon and the resulted Coulombic efficiency lower than 100% (Fig. S9) are largely due to the side reaction of oxygen evolution [30, 71]. The measurements were taken at unprecedented high rates of 200–400 C (50–100 A g^−1^). BGC-750 performs best throughout the whole rate range. Remarkably, maximum capacity of 257 mAh g^−1^ was obtained at starting rate of 2 C, and capacities of 126, 99 and 72 mAh g^−1^ were obtained at rates of 80 C, 200 C and 400 C, respectively. In addition, the gravimetric capacitances of BGCs and AC are calculated by integrating the enclosed area between discharge curves and the horizontal time axis (Fig. S10). The equation utilized is listed in the supporting information. These extreme current densities were never been employed in our previous zinc-air or compound cathodes-based zinc ion batteries [72, 73]. The capacities of the BGC-650 and BGC-850 cathodes are lower than those of BGC-750 with BGC-850 as the more inferior one. AC has the lowest surface area and degree of graphitic disorder. Yet the high content of microporosity (i.e., 87.83% of the total pore volume) of AC is expected to endow higher utilization of the surface area and heteroatomic/defective sites, because the wall of microporosity is probably more favorable for charge adsorption in capacitors than that of meso-/macroporosity [74, 75]. As a result, the capacities of AC are only slightly lower than those of BGC-850 at all current densities. Given the moderate content of heteroatom and degree of graphitic disorder for BGC-750, this comparison reveals that the surface area and porosity structure are more important in determining the specific capacities of the BGC cathode. This phenomenon to some extent indicates that the charge storage should largely be a surficial process, since the much higher surface area of BGC-750 (around twice of other BGCs) can expose much more active sites on the cathode surface. Also, the hierarchical porous structure consisted of open macropores, interconnected mesopores and macropores can effectively facilitate the ion diffusion, which endows the cathode with excellent capacity retention at the extreme rates [46, 47, 50, 76]. The more facile ion transport in BGC-750 can also be supported by the electrochemical impedance spectroscopy (EIS) analysis. Figure S11a shows the Nyquist plots of BGCs and AC, where the semicircular curves in the high-frequency region refer to the charge transfer resistance (*R*_ct_), while the slope lines in the low-frequency range link to the Warburg impedance (*Z*_W_) [36]. The equivalent circuit employed for the data analysis is shown in Fig. S11b, where *R*_e_ and CPE denote the total ohmic resistance and the constant phase element, respectively. The BGC-750 exhibits the smallest *R*_ct_ (67.6 Ω) than BGC-650 (71.3 Ω), BGC-850 (96.2 Ω) and AC (124.1 Ω). Moreover, BGC-750 curve exhibits a nearly vertical straight line at the ion diffusion controlled low-frequency range, which is reflective of the unimpeded ion transportation [48, 49].

It is instructive to compare the electrochemical performance of BGC-750 with that of the state-of-the-art carbon cathodes in hybrid zinc ion capacitors. Table S4 summarizes the properties of the reported carbon-based zinc ion capacitors in literature by far, including electrolyte, cell configuration, capacity, energy/power and cyclability. First, the rate performance of BGC-750 outperforms most existing carbon cathodes (Fig. 2d) [47–50, 64, 77–80]. After counting into the working potential, the Ragone plots of the BGCs and AC-based zinc ion capacitors are shown in Fig. 2e (The calculation methods used for calculation are in Supporting Information). The cell with BGC-750 cathode delivers a specific energy density of 168 Wh kg^−1^ at a power density of 327 W kg^−1^. At an extremely high power of 61,700 W kg^−1^, energy density of 45 Wh kg^−1^ still remains. According to the side-by-side comparison in Fig. 2f, this energy level is substantially superior to other carbon cathodes-based zinc ion capacitors, for instance activated carbon [64], chemical activated graphene [30], heteroatoms doping carbons [48, 50], hierarchical porous carbons [47, 49], polymer aerogels [80], etc.

An effective approach to understand the intrinsic kinetics of the charge storage in electrodes is to mathematically analyze the change of active current as a function of potential scan rate. The relationship between the two variables may be expressed as *i* = *av*^*b*^, where *i*, *v*, *a*, and *b* are on behalf of the current, scan rate and two adjustable constants [81]. The linear relationship (*b* = 0.5) indicates a diffusion limited process. Conversely, a *b* value of 1 originates from a surface capacitive behavior that dominates the charge storage process [82, 83]. The values of current (*i*) and scan rate (*v*) were read from the CV curves (Fig. S12), and the indicative *b* value can be represented by the slope of log *i* versus log *v* profiles (Fig. S13). The calculated *b* values of cathodic and anodic peaks of BGCs and AC are 0.91–0.99 and 0.85–0.92, respectively. These *b* values indicate that the charge storage processes in BGC cathodes are capacitive in nature occurring on the carbon surface with negligible ion diffusion action in carbon bulk, which agrees with the aforementioned observation. Meanwhile, the contribution of the capacitive charge storage was quantitatively calculated via dividing the current response into two parts (proportional to *v*^1/2^ and *v*) as following equation: *i* = *k*_1_*v* + *k*_2_*v*^1/2^ [63]. Variables *k*_1_ and *k*_2_ represent the proportions of the capacitive and non-capacitive contributions. As shown in Fig. S14, the capacitive contributions of BGC-650, -750 and -850 at a scan rate of 10 mV s^−1^ are 67.8%, 68.9% and 71.1%. In addition, the proportion of the capacitive contribution increases at high scan rates, exceeding 91% at 100 mV s^−1^ (Fig. 2g). The high proportion of capacitive charge storage is favorable for achieving high power AZBs [84, 85]. Furthermore, as shown in Fig. 2h, the BGCs cathodes also exhibit excellent cyclability upon extremely long cycling by virtue of such a capacitive ion storage process absent of bulk ion intercalation/extraction, which inevitably leads to volume change and interfacial resistance increase. Also, the GCD profiles for first three cycles are shown in Fig. S15.

### Charge Storage Mechanism Investigation and Active Sites Identification

**Fig. 3:**
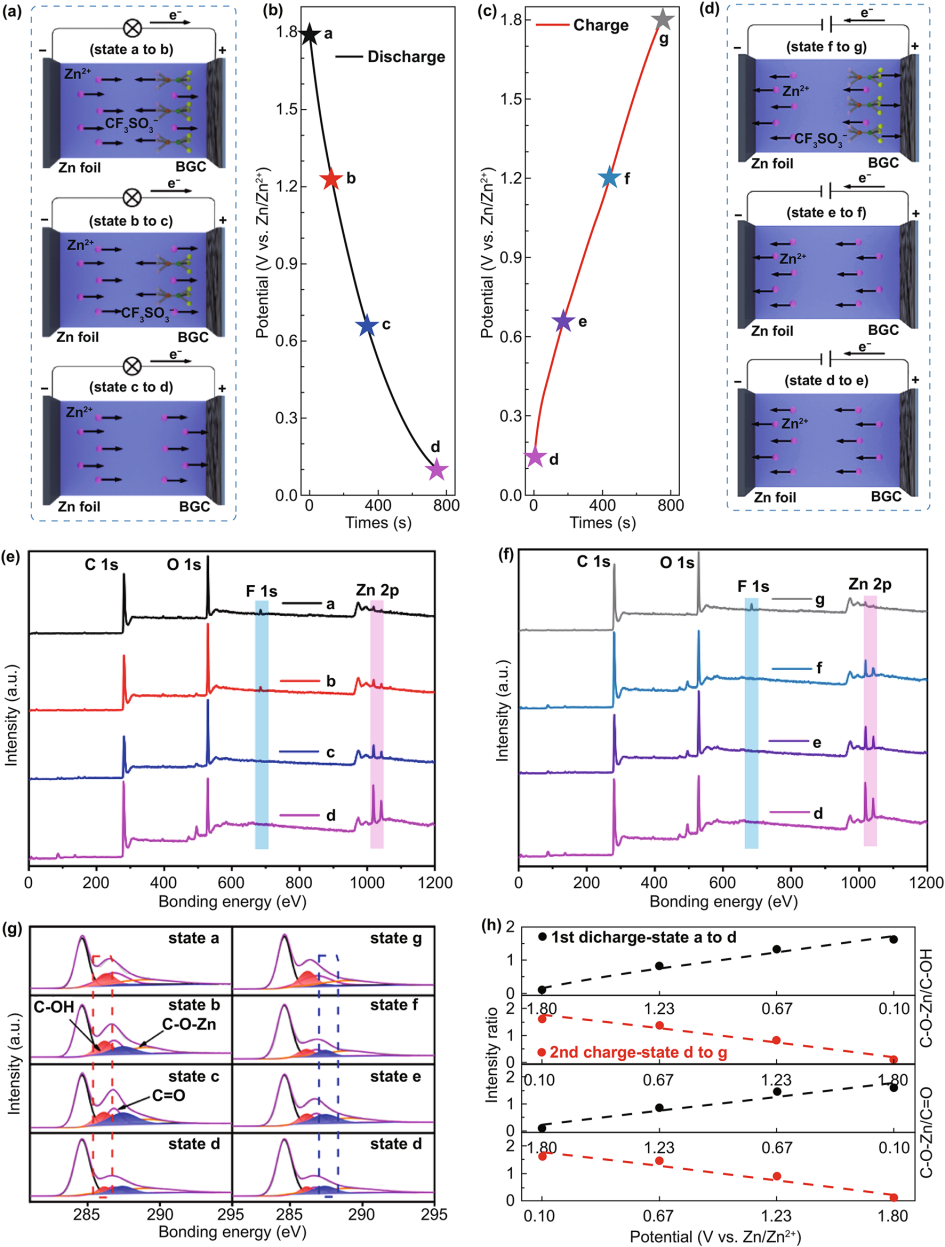
**a**, **d** Schematic diagram of the cation and anion transportation during discharge/charge. **b**, **c** The selected voltage points in the discharge/charge curves of BGC-750 for ex situ characterization. **e**, **f** Ex situ XPS spectra at the selected states. **g** Ex situ C 1*s* XPS spectra at the selected states. **h** The intensity ratio of C–O–Zn/C–OH and C–O–Zn/C=O peaks according to **g**

To explore the reasons for the electrochemistry and better understand the charge storage mechanisms of carbon cathodes in zinc ion capacitors, we performed systematic experiments and calculations to investigate the capacitive charge storage behavior of BGCs. First, XPS was employed to characterize the surface components at different charge and discharge states. To rule out the interference of fluorine in PVDF, we utilized sodium carboxymethyl cellulose (CMC) as the binder in the ex situ electrodes. Figure S16 displays the galvanostatic profiles and rate capacities of BGC-750 electrode using CMC binder. As comparing to Figs. 2c and S8b, the changing of binder from PVDF to CMC made negligible effect on the electrochemical performance of BGC-750. As shown in Fig. 3b, c, seven critical voltage points were selected in the first discharging and the second charging. Elements of C, O, F, S, N, Zn are detected in the XPS survey spectra, which exhibit distinct fluctuations in contents at different charge/discharge states. The signal of Zn 2*p* and F 1*s* is the descriptors of reflect to track the movement of Zn^2+^ cation and CF_3_SO_3_^−^ anion in the system. The typical open-circuit voltage of as-assembled zinc ion capacitor is around 1.1 V. As shown in Fig. 3e, upon the first discharge, the intensity of Zn 2*p* signal gradually increased from state *a* (full charge state, 1.8 V) to state *d* (full discharge state, 0.1 V), which clearly reveals the Zn^2+^ cation adsorption on BGC surface. The high intensity of Zn 2*p* in spectrum *d* indicates the large amount of Zn^2+^ accumulated on the cathode upon the deepest discharging. Meanwhile, the F 1*s* signal corresponding to the CF_3_SO_3_^−^ anion displays gradual attenuation upon discharge and thoroughly disappears at 0.67 V (point *c*). This phenomenon indicates the CF_3_SO_3_^−^ anions desorption from BGC surface. In the reverse charging process (Fig. 3f), the F 1*s* signal starts to reappear after point *f* (1.23 V) instead of from the primary *d*, *e* points. Therefore, the adsorption of CF_3_SO_3_^−^ anions is discovered to occur only at high voltage region. The attenuation of Zn 2*p* peaks starts from the lowest voltage of 0.1 V and lasts the whole 1.7 V voltage range, indicating that the Zn^2+^ desorption could occur at any potential. It is worth noting that trace Zn^2+^ (< 0.1 at%) is detected at full-charge states (*a* & *g*), which may be attributed to the residue Zn^2+^ trapped in carbon sub-surface. We also conducted the same ex situ characterization on the AC baseline. The resulted XPS survey spectra are shown in Fig. S17. The trends of increasing in Zn^2+^ signal upon discharging (*a* to *c* to *d*) and decreasing in Zn^2+^ signal upon charging (*d* to *e* to *g*) agree well with the observation in Fig. 3e, f, proving the reversible Zn^2+^ ions adsorption on AC. The very weak F 1*s* signals in the fully charge states of *a* and *g* indicate the restricted anion adsorption in AC. Considering the large spatial dimensions of CF_3_SO_3_^−^ anion [86] and the majority of microporosity with size below 1 nm in AC, it is expected that there is severe steric hindrance for CF_3_SO_3_^−^ anion diffusion within microporosity which increases the voltage polarization of CF_3_SO_3_^−^ adsorption and leads to very small amount of anion adsorption on AC at cutoff voltage of 1.8 V. We further analyzed the high-resolution C 1*s* spectra of BGC-750 to understand the possible charge transfer process involved in the aforementioned adsorption/desorption (Fig. 3g). Notably, the spectra show a pronounced C–O–Zn bonding component at 287.4 eV, which is resulted from the Faradic reaction between Zn^2+^ and C–OH or C=O [48, 87, 88]. The evolution of the peak intensity ratio of C–O–Zn to C–OH and C–O–Zn to C=O was evaluated and is plotted in Fig. 3h. The data indicate the increased C–O–Zn bonds and decreased C–OH/C=O groups upon discharge, and this well-defined trend is reversible in the charge process. This observation reveals a representative oxygen groups involved charge transfer process that contributes capacity. More comprehensive analysis was conducted by the aid of first principle calculation as shown below.

Based on the preceding analyses, we claim that the BGC cathode undergoes a reversible dual-ion adsorption process in zinc ion capacitors. Both the cation and anion act as charge carriers for energy storage, yet they function at different voltage regions (Fig. 3a, d). As discharging from 1.8 to 1.23 V, the desorption of anion and the adsorption of cation take place simultaneously. As discharged below 0.67 V, the anion desorption completes together with the continuing cation adsorption. The charge storage below 0.67 V is simply responsible by the cations. In the charging process, the dual-ion adsorption follows the opposite path of the discharging. The majority of the cation desorption distributes in the low voltage. The anion adsorption occurs only as the cathode enters the high voltage region.

**Fig. 4:**
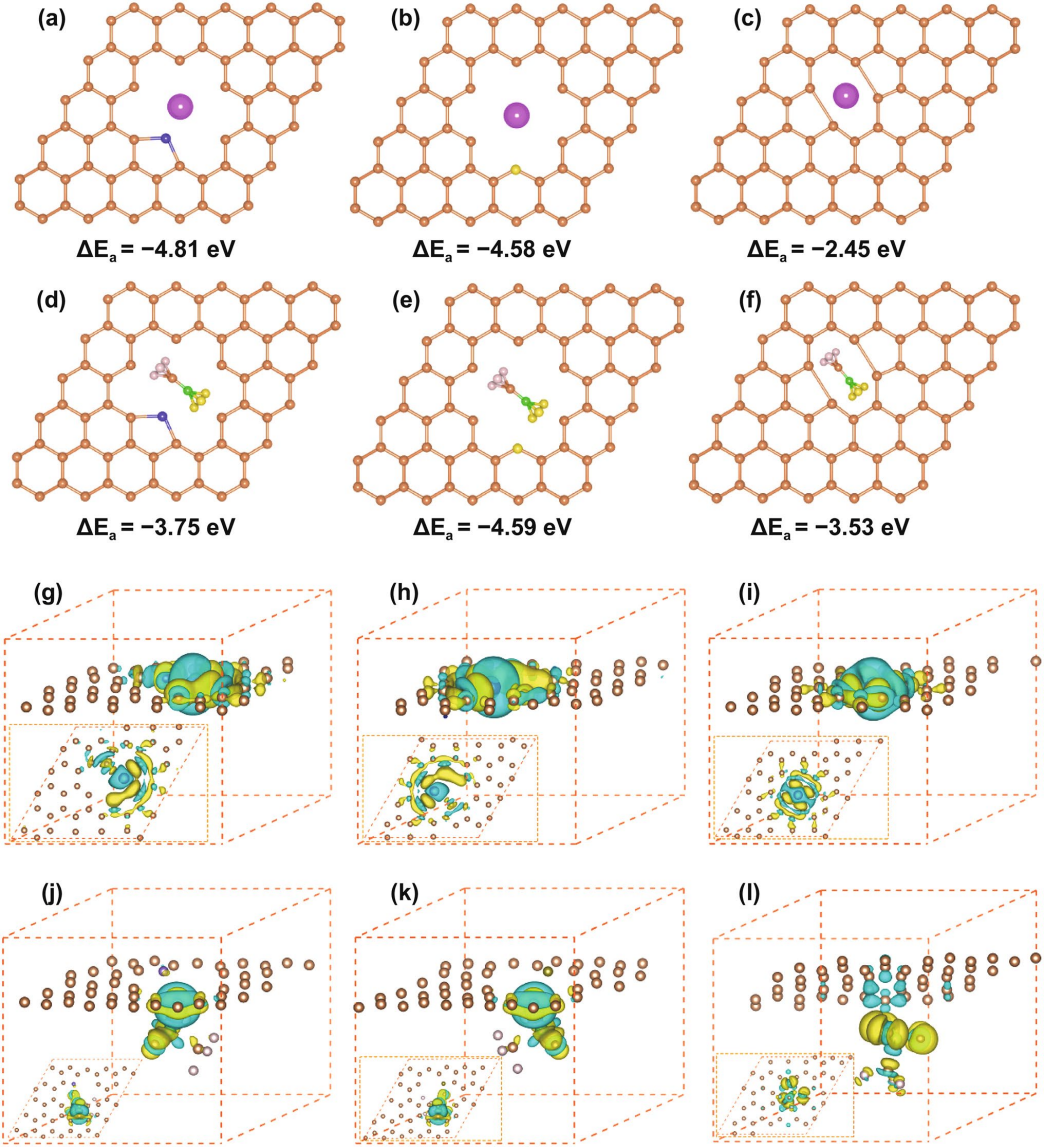
Theoretical simulations of Zn^2+^/CF_3_SO_3_^−^-adsorption on different graphitic structures. The configurations and corresponding adsorption energy values of single Zn^2+^/CF_3_SO_3_^−^ adsorbed at **a**/**d** N-5, **b**/**e** O-II sites and **c**/**f** divacancy sites. Side and top views (inserts) of electron density differences of Zn^2+^/CF_3_SO_3_^−^ absorbed in the **g**/**j** N-5, **h**/**k** O-II sites and **i**/**l** divacancy defect sites. Yellow and blue areas represent the increased and decreased electron density, respectively. Brown, purple, light yellow, green, gray, pink and blue balls represent C, N, O, S, F, Zn, and H atoms, respectively. The iso-surfaces are the 0.002 electron bohr^3^

To determine the specific active sites for the dual-ion adsorption in the BGC cathodes, we calculated the relative adsorption energy (Δ*E*_a_) values of Zn^2+^ cation and CF_3_SO_3_^−^ anion at the atomic heterogeneous sites and defective sites as comparing to that of on flawless graphene surface (Table S5). As revealed by XPS, Raman and BET results, BGCs are rich in heteroatoms and defects, the populations of which are significantly amplified by the immense surface areas. For the heteroatom sites, we choose nitrogen-5, -6 and oxygen-I, -II as the structure models. For the lattice defects, a representative defect of divacancy is employed for calculation [89, 90]. An individual Zn^2+^ or CF_3_SO_3_^−^ ion was placed close to each site, and the optimized geometry structure is ascertained in light of the calculated Δ*E*_a_. As shown in Fig. 4a, d, the Δ*E*_a_ values of Zn^2+^ and CF_3_SO_3_^−^ at N-5 are − 4.81 and − 3.75 eV, respectively, which are much higher than those on flawless graphene surface (− 0.02 eV, − 1.29 eV, Fig. S18c, f). This indicates that N-5 is active toward both cation and anion adsorption. Similar ion affinity is also observed for N-6 site (Fig. S18a, d). It was reported that C–O–C-type groups are active in chemically adsorbing various alkali ions in organic systems [91–93]. Our calculation result indicates that these O-II sites are also capable for Zn^2+^ cation adsorption in aqueous system (Δ*E*_a_ = − 4.58 eV). Remarkably, the negative charged CF_3_SO_3_^−^ ion also exhibits a high Δ*E*_a_ at O-II sites (Δ*E*_a_ = − 4.59 eV). Certain electron-rich part of the CF_3_SO_3_^−^ molecular may be functional for this interaction. In addition, the Δ*E*_a_ remains considerably high as Zn^2+^/CF_3_SO_3_^−^ was placed around C=O-type groups (O-I) (Fig. S18b, e). Divacancy is a typical lattice defect in carbon, which is resulted from the missing of two neighboring carbon atoms [90, 94, 95]. The divacancy defects can significantly generate electron redistribution, thus adsorbing the charged ions. For instance, the Δ*E*_a_ of Zn^2+^ and CF_3_SO_3_^−^ at the divacancy sites are − 2.45 and − 3.53 eV, respectively, higher than the flawless graphene surface baseline (Fig. 4c, f). To confirm the formation of bonding between surficial sites and ions, we conducted the differential charge density analyses for the adsorption configurations. The increase of electron density in the intermediate domain between graphene sheet and ion is reflective of the charge transfer between the surficial sites and ions. As shown in Figs. 4g–l and S18g-l, there are net increases in charge density for both heteroatom (N-5/-6, O-I/-II) and divacancy sites, indicating occurrence of the chemical adsorption processes involving in Faradic charge transfer [96–98]. The comprehensive calculations reveal the capacitive dual-ion adsorption at the heteroatom moieties and lattice defects in BGCs, providing insight of the significantly improved AZB energy and power by virtue of advanced charge storage mechanism.

### Electrochemical Performance of Flexible Pouch-/Cable-type AZB Devices

**Fig. 5:**
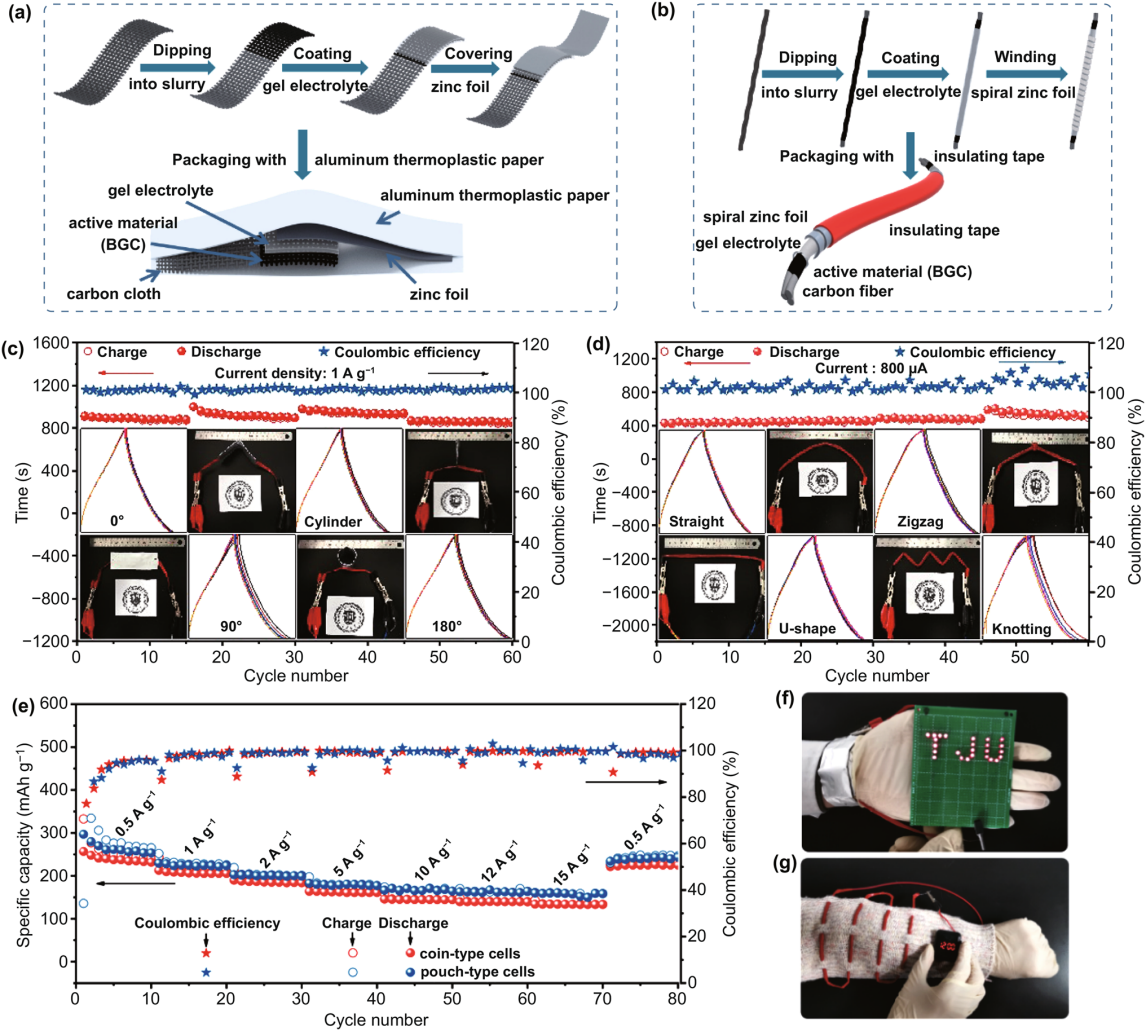
**a**, **b** Schematic diagrams of pouch-type and cable-type quasi-solid-state zinc ion capacitors. Cycling performance of pouch-type cell **c** and cable-type cell **d** upon deformation. The inserts exhibit the optical images of the deformed cells and corresponding galvanostatic charge–discharge profiles. **e** Specific capacities at various current densities for coin cells and pouch-type cells. **f** Photograph of LED array powered by two pouch-type cells in series. **g** Photograph of a digital watch powered by four cable-type cells in series

One critical target of developing AZBs is to power the flourishing flexible and wearable electronic devices [47]. To verify the applicability of the BGC-based zinc ion capacitors as flexible power sources, we go steps further than the routine coin cell and fabricate quasi-solid-state pouch and cable-type cells. Figure 5a, b demonstrates the procedures of the pouch-type and cable-type cells fabrication. We first developed a gel polymer electrolyte (GPE) with PVA as the gel matrix and ZnSO_4_ as a neutral salt. According to our previous experience, GPE-based zinc-air batteries could be easily deteriorated by the fast water loss due to the open cell structure [99–101]. The cells in this work are free of this issue, because the PVA/ZnSO_4_ gel electrolyte performs steadily in the closed cells. For the pouch cells (Fig. 5a), the BGC-750 anchored carbon cloth, and the freestanding GPE film and zinc foil were layer-by-layer assembled into a sandwich-type structure followed by the air evacuation and firm packaging. The cable-type cell has a coaxial structure with BGC-750-loaded carbon fiber as the core and GPE/spiral zinc foil as the shells (Fig. 5b). Superior to the typical zinc wire-centered zinc-air cells, the cathode cores in our setups are well protected by the GPE/zinc metal shells; hence, the typical active material loss is totally eliminated. The as-prepared pouch- and cable-type cells exhibit excellent flexibility upon various deformations without apparent sacrifice of electrochemical performance. For instance, the pouch-type cell can be easily bent 90°, rolled up to hollow circular column and folded 180°, at which states the cell maintains the pristine flat-shape electrochemistry in terms of voltage profiles, capacity and CE (Fig. 5c). Also for the cable-type cell (Fig. 5d), the deformation of U-shape curving, zigzag bending and even tightly knotting barely affect the performance of the cable-type cell, as revealed by the mostly overlapped GCD profiles. In addition, due to the practical mass loading and shape flexibility for fulfilling different application conditions, the pouch-type cell is the best configuration for evaluating the true practicability of a new battery system. Therefore, we also collected the specific capacities of the pouch-type cells at rate of 2–60 C. The corresponding energy/power densities were calculated and were compared with the coin cells side-by-side. The resulted data are plotted in Figs. 5e and S19. Remarkably, the two cell configurations display comparable performance. The slightly higher capacities of the pouch-cell are probably benefited from the commercial battery-level carbon cloth as the current collector which is better at minimizing the active particle agglomeration than the flat stainless steel foils in coin cell. Figure 5f, g demonstrates two samples of utilizing the pouch- and cable-type cells for wearable electronic devices. The pouch-type cell can be twisted around human wrists to power other wearable electronics (lighting LED bulbs as an example). The cable-type cells can be easily connected in series or parallel to multiply the voltage or current, which can be knitted into clothes for powering smart watches and other equipment.

## Conclusion

To summarize, we employed a unique dual-ion adsorption mechanism in the carbon cathode for aqueous zinc-based batteries. Experiments and DFT calculations reveal that both the cation and anion function as charge carriers and are reversibly adsorbed at the heteroatom moieties and lattice defects on the carbon surface. The optimized BGC cathode possesses immense surface area, hierarchical porosity and defect-rich graphite tissue and hence maximizes the population of the electrochemically active sites for the reversible dual-ion adsorption. Quantitative analysis indicates that the dual-ion adsorption process is primarily contributed by capacitive charge storage and thus is kinetically facile. This enables the aqueous zinc-based battery to deliver unrivaled combination of high energy and power characteristics, reaching 168 Wh kg^−1^ and 61,700 W kg^−1^, respectively. The BGC-based zinc ion capacitors are also extended to quasi-solid-state pouch- and cable-type configurations. The flexible cells exhibit high reliability under various deformation conditions, serving as promising power sources for the wearable devices.

## Supplementary Information

Below is the link to the electronic supplementary material.

Supplementary Information 1 (PDF 2424 kb)
