# Supplementary material for: Identifying Heteroatomic and Defective Sites in Carbon with Dual-Ion Adsorption Capability for High Energy and Power Zinc Ion Capacitor
Source: Nanomicro Lett. 2021 Jan 21;13:59. doi: 10.1007/s40820-021-00588-5 (PMC8187496; doi:10.1007/s40820-021-00588-5)
Supplement: Supplementary file 1 — Supplementary Information 1 (PDF 2424 kb) [file 40820_2021_588_MOESM1_ESM.pdf]

Supporting Information for

Identifying heteroatomic and defective sites in carbon with dual-ion adsorption capability for high energy and power zinc ion capacitor

Wenjie Fan[1,2], Jia Ding[1,]*, Jingnan Ding[1], Yulong Zheng[2], Wanqing Song[1], Jiangfeng Lin[1], Caixia Xiao[1], Cheng Zhong[1,]*, Huanlei Wang[2,]* and Wenbin Hu[1,]*

[1] Key Laboratory of Advanced Ceramics and Machining Technology (Ministry of Education), School of Materials Science and Engineering, Tianjin University, Tianjin 300072, People's Republic of China.

* Corresponding authors.

E-mail: jiading@tju.edu.cn (J. Ding), cheng.zhong@tju.edu.cn (C. Zhong), wbhu@tju.edu.cn (W. Hu)

[2] School of Materials Science and Engineering, Ocean University of China, Qingdao 266100, People's Republic of China.

* Corresponding authors.

E-mail: huanleiwang@gmail.com (H. Wang)

Supplementary Tables and Figures

S1. Experimental Section

S1.1 Electrochemical measurement

For zinc ion capacitors measurement, a CR2032 coin-type cell was chosen to test the electrochemical performance. The cathodes were prepared by coating the active material slurry on the stainless steel foil and drying at 80 °C for 12 h. The slurry is prepared by mixing BGCs (80 wt%), super P (10 wt%), polyvinylidene fluoride binder (10 wt%) in 1-methyl-2-pyrrolidinone. The mass loading of the active materials was about 1 mg cm^{-2}. The zinc metal foils polished with finegrained sandpaper were used as anodes. The thickness of the zinc foil is 0.5 mm. Filter paper was used as separator. 3 M $Zn(CF_3SO_3)_2$ aqueous solution (1 M refers to 1 mole of solute per kilogram deionized water) was used as electrolyte. The electrochemical performance including cycling voltammetry (CV), galvanostatic charge-discharge (GCD), electrochemical impedance spectroscopy (EIS) were tested on Chenhua electrochemical workstation (CHI760E, Shanghai, China) at room temperature. Both the CV and GCD were collected at a voltage range of 0.1-1.8 V. EIS was measured in the frequency range of 0.001-100000 Hz. Moreover, the specific capacity (C_1, mAh g^{-1}), specific capacitance (C_2, F g^{-1}), energy density (E_1, Wh kg^{-1}) and power density (P_1, W kg^{-1}) were calculated through the following equations:

$$C_1 = I \cdot \Delta t / 3.6 \quad (1)$$

$$C_2 = 2I \int V \cdot dt / V^2 m \quad (2)$$

$$E_1 = \int V \cdot I \cdot dt / 3.6m \quad (3)$$

$$P_1 = 3600E / \Delta t \quad (4)$$

where I, Δt, m and V are current, charge/discharge time, the mass of active materials on cathode and discharge voltage.

S1.2 Quasi-solid-state zinc ion capacitors fabrication

The gel electrolyte was prepared by adding 3.22 g $ZnSO_4$, 2.54 g LiCl and 2 g PVA (M. W. 300000) in 20 ml deionized water at 80 °C and stirring the solution for 1 h. For the assembly of pouch-type cells (2 × 5 cm, thickness: 4 mm), a piece of carbon cloth (1 × 4 cm, thickness: 0.32 mm, Cetech Co., Ltd., Taiwan) loaded with active material slurry was dried at 80 °C for 12 h and then covered with gel electrolyte followed by an age step in refrigerator at -10 °C for 6 h. The treated carbon cloth (active materials loading: 1 mg cm^{-2}) and zinc foil (1 × 4 cm, thickness: 0.5 mm) were superimposed together and then sealed with aluminum thermoplastic paper. The cable-type cells (diameter: 3 mm, length: 15 cm) were assembled by winding the treated carbon fiber (diameter: 1.5 mm, mass loading: 0.2 mg cm^{-1}) with spiral zinc foil (width: 2 mm) and sealing with insulating tape. The electrochemical performance of the quasi-solid-state zinc ion capacitors was measured in a battery test system (Land, CT2001A, China) at room temperature.

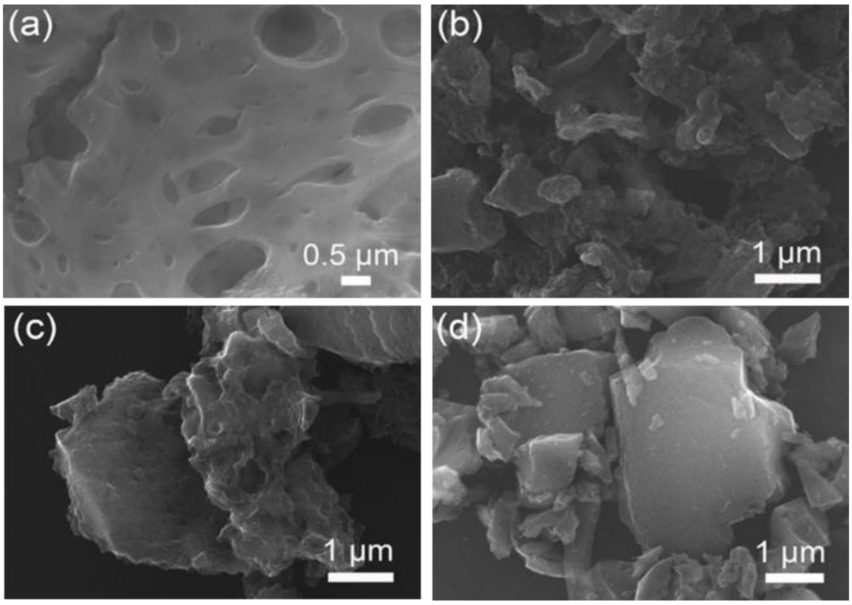

Fig. S1 SEM micrographs of **a** BGC-750, **b** BGC-650, **c** BGC-850, **d** AC

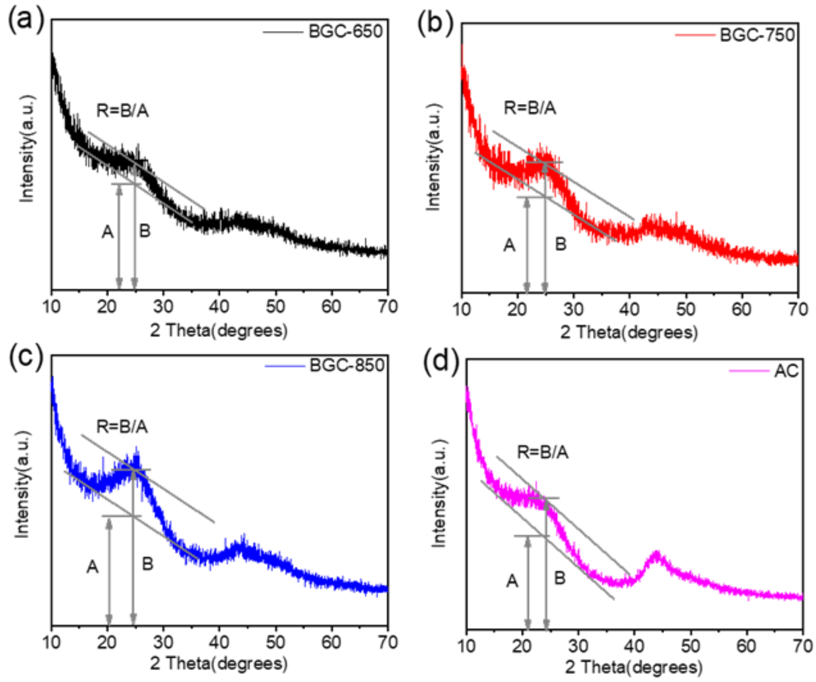

Fig. S2 The calculation method of *R* value for **a** BGC-650, **b** BGC-750, **c** BGC-850 and **d** AC

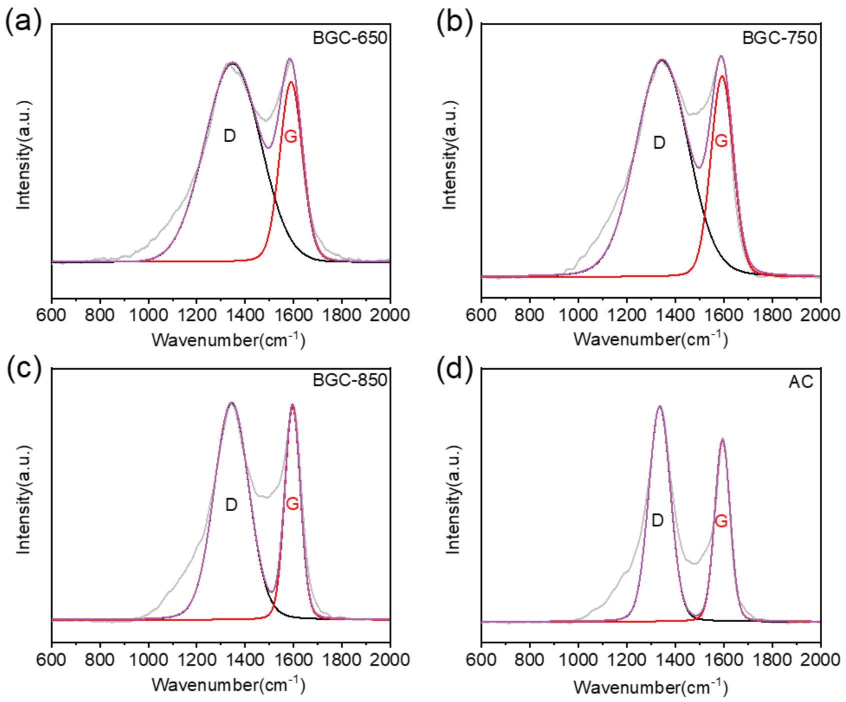

Fig. S3 Fitted Raman spectra of **a** BGC-650, **b** BGC-750, **c** BGC-850, **d** AC using Voigt function

Table S1 Physical and chemical parameters for the bone glue derived carbons (BGCs) and activated carbon

Sample	S_{BET} (m^2 g^{-1})[a]	V_t (cm^3 g^{-1})[b]	Pore volume (%)		R	I_D/I_G	XPS composition (at. %)		
			$V < 2$ nm	$V > 2$ nm			C	O	N
BGC-650	1723.19	0.797	50.38	49.62	1.27	2.62	85.82	9.88	4.30
BGC-750	3657.45	2.428	23.86	76.14	1.43	2.54	92.00	5.72	2.28
BGC-850	1493.60	1.061	22.35	77.65	1.48	2.27	90.95	7.94	1.11
AC	1263.35	0.541	87.83	12.17	1.52	1.68	91.07	8.93	0

[a] Surface area was calculated by BET method.

[b] The total pore volume was determined by DFT method.

Table S2 Relative surface concentrations (%) of nitrogen and oxygen species obtained by fitting N 1s and O 1s core level XPS spectra

Samples	N-6	N-5	N-Q	N-X	O-I	O-II	O-III
BGC-650	12.00	43.53	25.35	19.12	38.26	37.20	24.54
BGC-750	14.30	40.81	26.82	18.07	40.05	36.75	23.20
BGC-850	28.59	28.84	31.38	11.19	40.66	39.48	19.86
AC	-	-	-	-	46.75	37.47	15.78

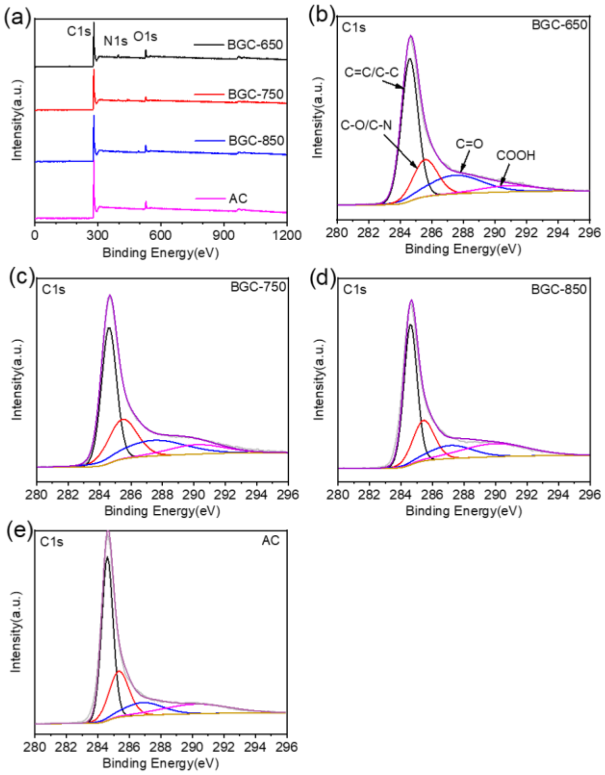

Fig. S4 a XPS survey spectra of BGCs and AC. High-resolution XPS C 1s spectra of **b** BGC-650, **c** BGC-750, **d** BGC-850, and **e** AC

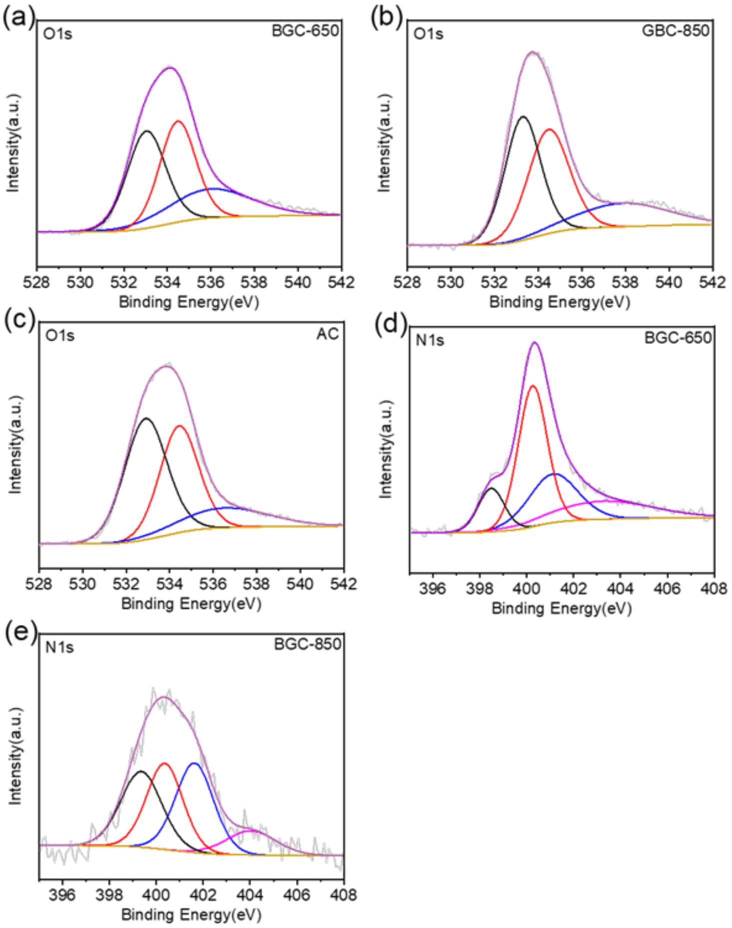

Fig. S5 High-resolution XPS O 1s spectra of **a** BGC-650, **b** BGC-850, **c** AC. High-resolution XPS N 1s spectra of **d** BGC-650, **e** BGC-850

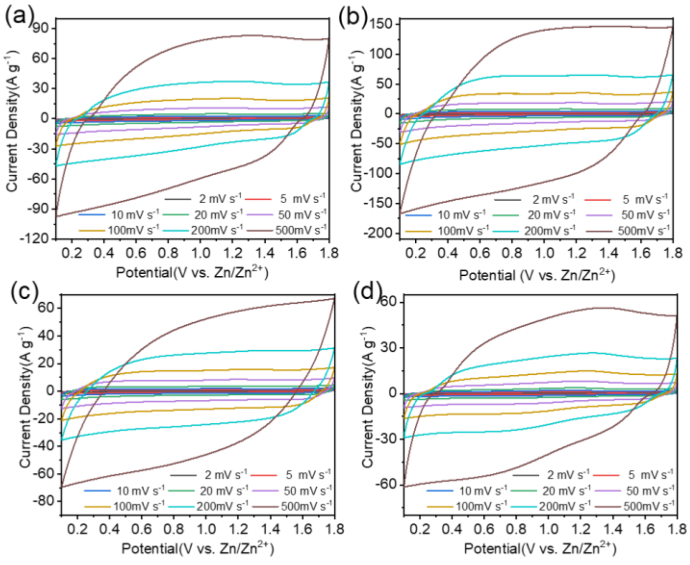

Fig. S6 CV curves of **a** BGC-650, **b** BGC-750, **c** BGC-850 and **d** AC based zinc ion capacitors at scan rates range of 2-500 mV s^{-1}

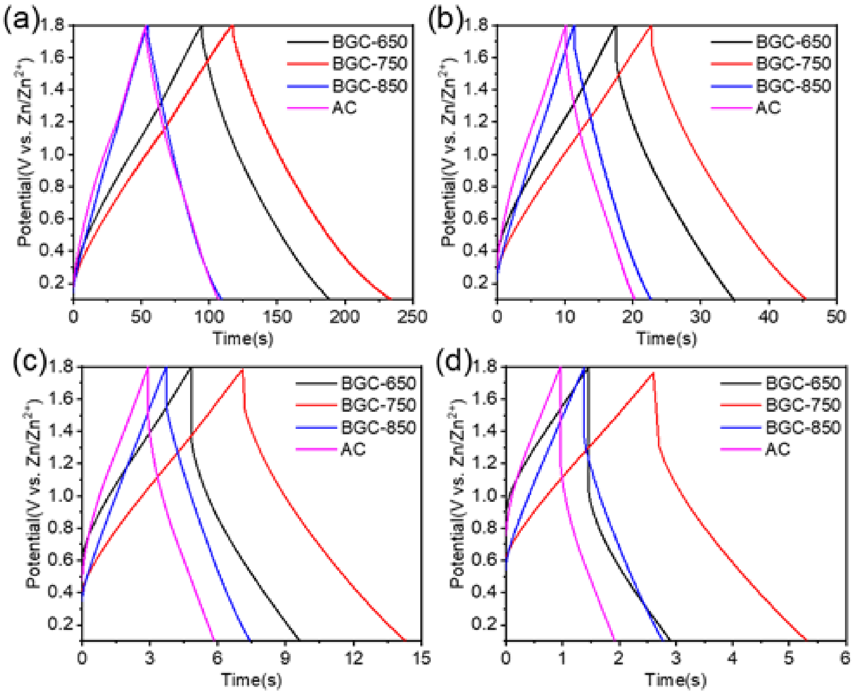

Fig. S7 Galvanostatic charge-discharge profiles of BGCs and AC based zinc ion capacitors tested at **a** 5 A g^{-1}, **b** 20 A g^{-1}, **c** 50 A g^{-1} and **d** 100 A g^{-1}

Table S3 The IR drops of the BGCs and AC electrodes at the current densities from 50 A g^{-1} to 100 A g^{-1}

Current Density	BGC-650	BGC-750	BGC-850	AC
50 A g^{-1}	0.420	0.270	0.217	0.316
60 A g^{-1}	0.485	0.309	0.254	0.370
70 A g^{-1}	0.552	0.336	0.291	0.424
80 A g^{-1}	0.606	0.380	0.329	0.478
90 A g^{-1}	0.668	0.413	0.368	0.531
100 A g^{-1}	0.731	0.494	0.405	0.584

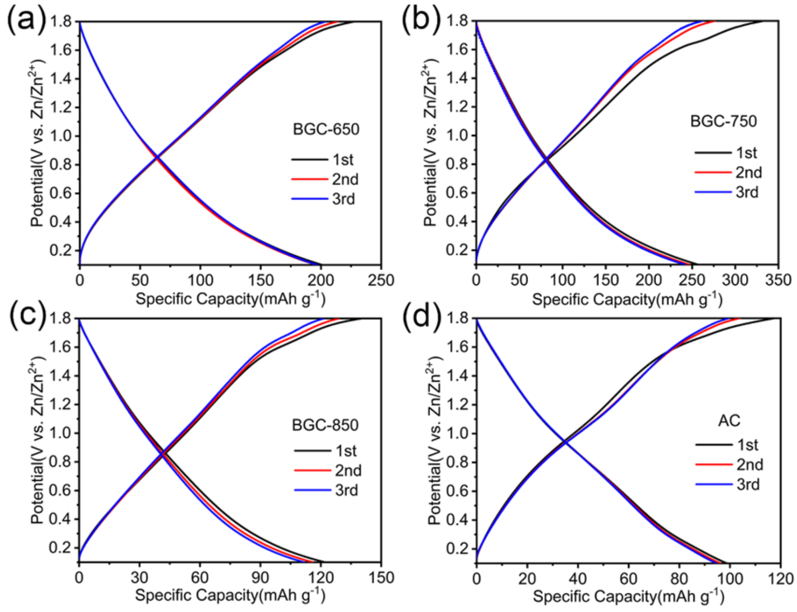

Fig. S8 Galvanostatic charge-discharge profiles for first three full voltage window (0.1-1.8 V) cycles for **a** BGC-650, **b** BGC-750, **c** BGC-850, and **d** AC based zinc ion capacitor at 0.5 A g^{-1}

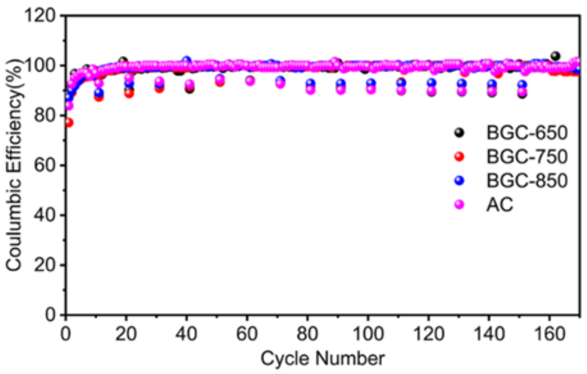

Fig. S9 The Coulombic efficiency of BGCs and AC based zinc ion capacitor

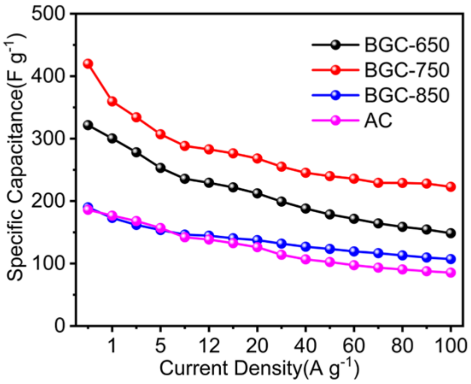

Fig. S10 Specific capacitances at various current densities of BGCs and AC

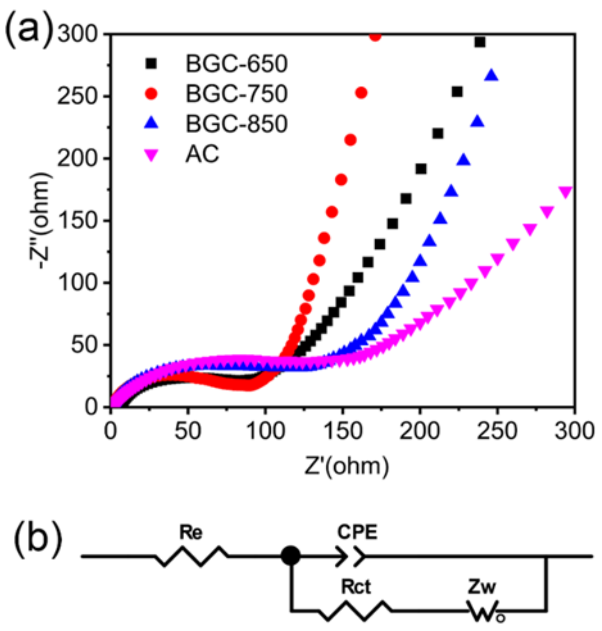

Fig. S11 a Nyquist plots of BGCs and AC based zinc ion capacitors. **b** The corresponding simulating equivalent circuit

Table S4 Electrochemical performance comparison for reported carbon-based zinc ion capacitors

**Cathode** (Specific surface area)	**Anode**	**Electrolyte (Voltage)**	**Cell configuration**	**Capacity**	**Energy and power density**	**Capacity retention**	**Ref. # Publish year**
BGC (3657.5 m^2 g^{-1})	Zn foil	3 M $Zn(CF_3SO_3)_2$ (aq.) (0.1-1.8 V)	CR2032	257 mA h g^{-1} (0.5 A g^{-1}) 76 mA h g^{-1} (100 A g^{-1})	168 Wh kg^{-1} 61696 kW kg^{-1}	--	This work
		PVA gel electrolyte (1 M $ZnSO_4$) (0.1-1.8 V)	Pouch type cell	297 mA h g^{-1} (0.5 A g^{-1}) 159 mA h g^{-1} (15 A g^{-1})	182 Wh kg^{-1} 10419 W kg^{-1}	--	
Porous carbon (523 m^2 g^{-1})	Zn on carbon cloth	Gelatin gel electrolyte (1 M $ZnSO_4$) (0.2-1.8 V)	Two-electrode sandwich-supercapacitor	132.7 mA h g^{-1} (0.2 A g^{-1}) 54.5 mA h g^{-1} (4 A g^{-1})	82.4 Wh kg^{-1} 3.76 kW kg^{-1}	87.6% after 10000 cycles at 1 A g^{-1}	[S1] 2020
aMEGO (2957 m^2 g^{-1})	Zn foil	3 M $Zn(CF_3SO_3)_2$ (aq.) (0-1.9 V)	CR2032	--	106.3 Wh kg^{-1} 31.4 kW kg^{-1}	93% after 80000 cycles at 8 A g^{-1}	[S2] 2019
2D porous carbon (597 m^2 g^{-1})	Zn foil	1 M $ZnSO_4$ (aq.) (0.2-1.8 V)	Pouch type cell	116.8 mA h g^{-1} (0.5 A g^{-1}) 55.4 mA h g^{-1} (20 A g^{-}	86.8 Wh kg^{-1} 12.1 kW kg^{-1}	81.3% after 6500 cycles at 5 A g^{-1}	[S3] 2019

				^{1})			
N doping porous carbon (2762.7 m^2 g^{-1})	Zn foil	1 M $ZnSO_4$ (aq.) (0-1.8 V)	Two electrode system	177.8 mA h g^{-1} (4.2 A g^{-1})<br>108.2 mA h g^{-1} (33.6 A g^{-1})	107.3 Wh kg^{-1} 24.9 kW kg^{-1}	73.6% after 100000 cycles at 16.7 A g^{-1}	[S4] 2019
rGO/CNT hybrid fiber (265 m^2 g^{-1})	Zn/graphite fiber	PAA gel electrolyte (2 M $ZnSO_4$) (0-1.8 V)	Three electrode system	104.5 F cm^{-3} (400 mA cm^{-3})<br>76.7 F cm^{-3} (8000 mA cm^{-3})	^{a}49 mWh cm^{-3}<br>3599 mW cm^{-3}	98.5% after 10000 cycles at 3200 mA cm^{-3}	[S5] 2019
Biomass derived carbon (3401 m^2 g^{-1})	Zn foil	2 M $ZnSO_4$ with 1 M Na_2SO_4 (aq) (0-1.8 V)	CR2025	305 mA h g^{-1} (0.1 A g^{-1})<br>101 mA h g^{-1} (5 A g^{-1})	118 Wh kg^{-1}<br>3.158 kW kg^{-1}	94.9% after 20000 cycles at 2 A g^{-1}	[S6] 2019
Hollow carbon spheres (819.5 m^2 g^{-1})	Zn on carbon cloth.	Polyacrylamide hydrogel electrolyte (0.15-1.95 V)	Two-electrode sandwich-supercapacitor	86.8 mA h g^{-1} (0.5 A g^{-1})<br>47.1 mA h g^{-1} (4 A g^{-1})	59.7 Wh kg^{-1}	98% after 15000 cycles at 1 A g^{-1}	[S7] 2019
PPy<br>(Not reported)	Zn/graphite paper	2 M $ZnSO_4$ (aq.) and 3 M NH_4Cl (aq.)<br>(0.6-1.6 V)	Two electrode system	151.1 mA h g^{-1} (0.5 A g^{-1})<br>87.6 mA h g^{-1} (16 A g^{-1})	^{b}119 Wh kg^{-1}<br>11.7 kW kg^{-1}	76.7% after 1000 cycles at 8 A g^{-1}	[S8] 2018
Graphene@PANI<br>(Not reported)	Zn foil	2 M $ZnSO_4$ (aq.)<br>(0.3-1.6 V)	Two-electrode sandwich-supercapacitor	154 mA h g^{-1} (0.1 A g^{-1})<br>106 mA h g^{-1} (5 A g^{-1})	205 Wh kg^{-1}<br>2.455 kW kg^{-1}	80.5% after 6000 cycles at 5 A g^{-1}	[S9] 2018
AC<br>(1923 m^2 g^{-1})	Zn foil	2 M $ZnSO_4$ (aq.)<br>(0.2-1.8 V)	CR2032	121 mA h g^{-1} (0.1 A g^{-1})<br>41 mA h g^{-1} (20 A g^{-1})	84 Wh kg^{-1}<br>14.9 kW kg^{-1}	91% after 10000 cycles at 1 A g^{-1}	[S10] 2018

[a] Calculated based on total volume of two electrodes and gel electrolyte.

[b] Calculated based on total active mass of both cathode and anode.

(The other data are calculated based on the mass of cathode materials.)

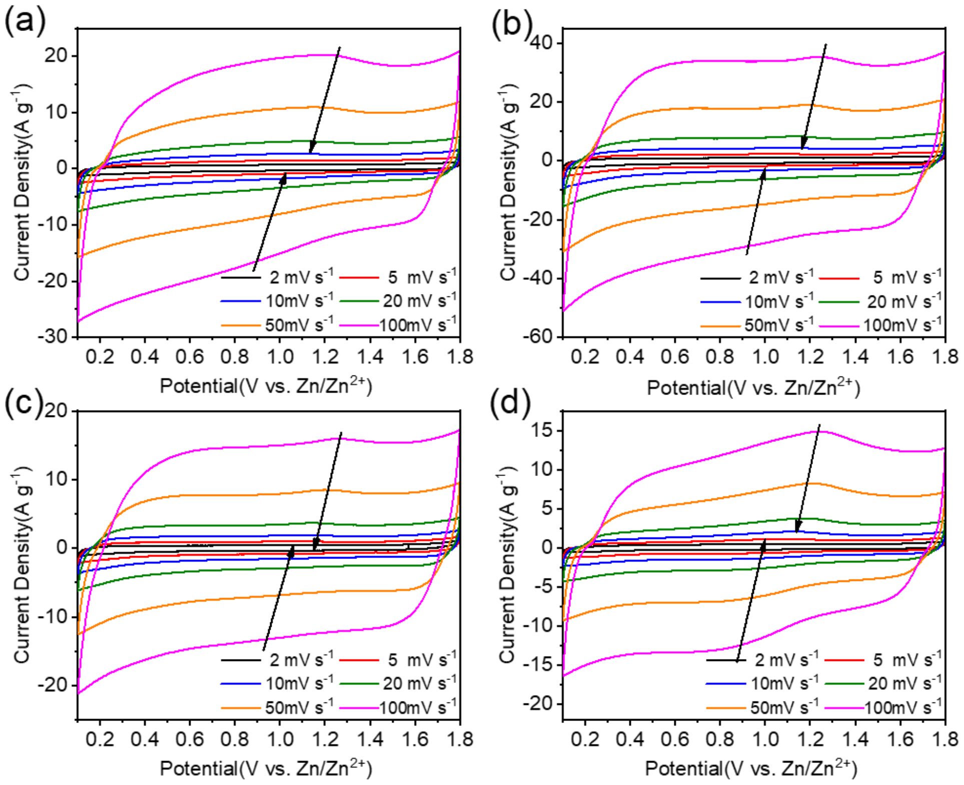

Fig. S12 CV curves **a** BGC-650, **b** BGC-750, **c** BGC-850 and **d** AC based zinc ion capacitors at scan rates range of 2-100 mV s^{-1}

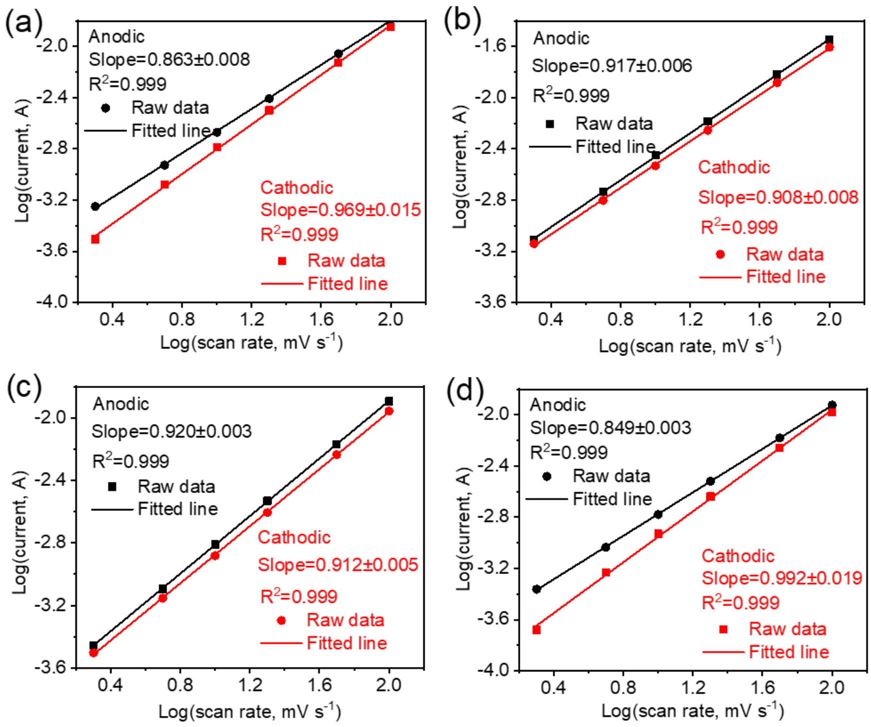

Fig. S13 Selected cathodic and anodic *b* values of peak currents (as indicated in Fig. S12) for **a** BGC-650, **b** BGC-750, **c** BGC-850 and **d** AC

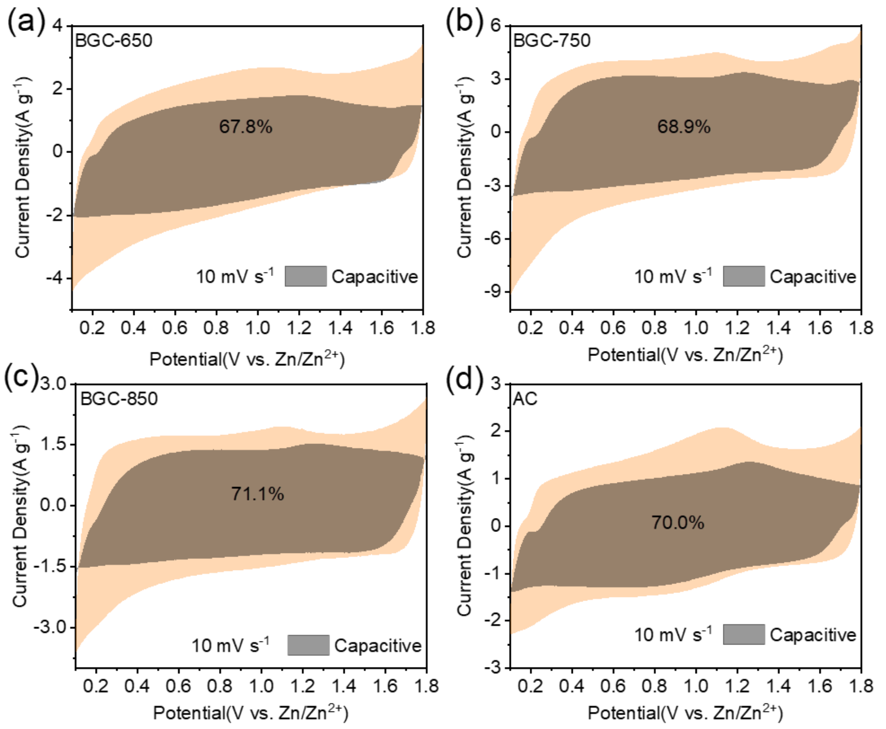

Fig. S14 The capacitive contribution of **a** BGC-650, **b** BGC-750, **c** BGC-850 and **d** AC cathodes based zinc ion capacitors at scan rate of 10 mV s^{-1}

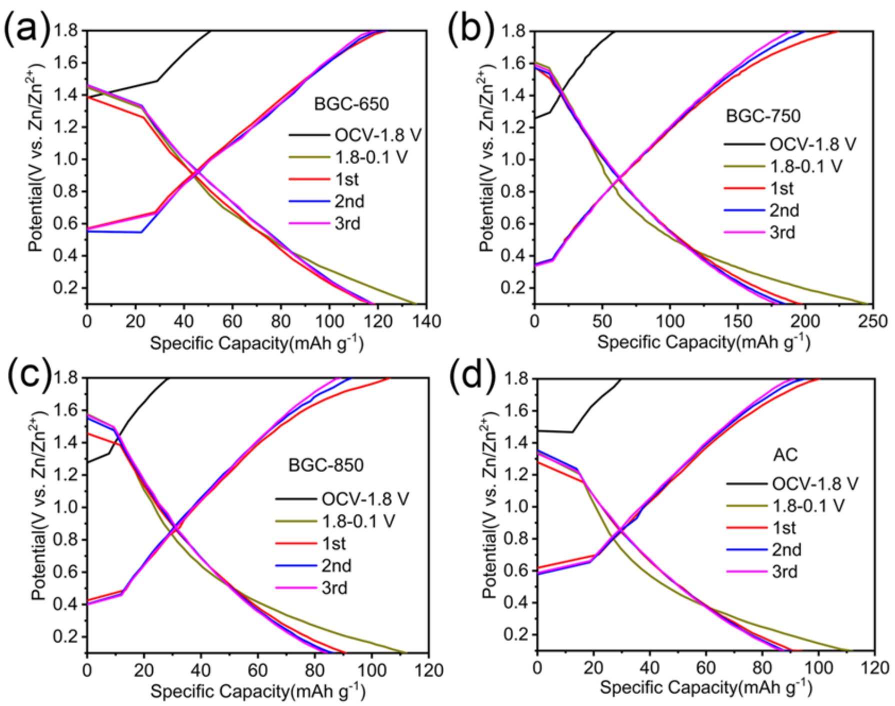

Fig. S15 Galvanostatic charge-discharge profiles of the initial several charge and discharge processes for **a** BGC-650, **b** BGC-750, **c** BGC-850, and **d** AC based zinc ion capacitor at 5 A g^{-1}

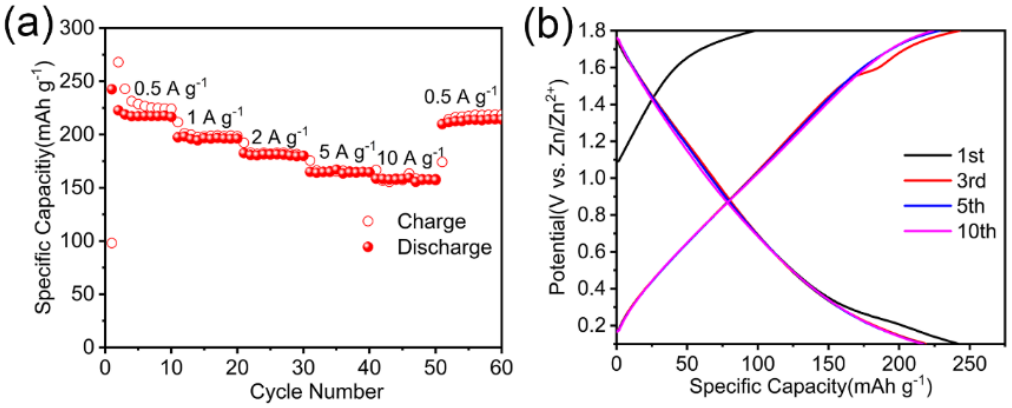

Fig. S16 a Specific capacities at various current densities and **b** galvanostatic charge-discharge profiles of BGC-750 electrode employing CMC binder

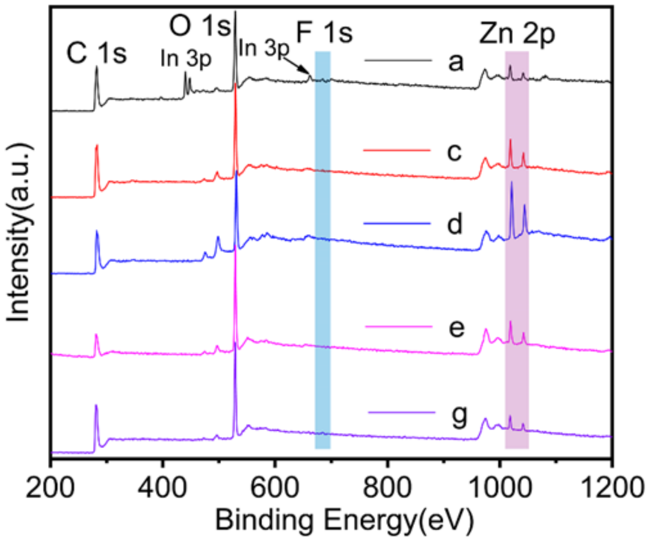

Fig. S17 *Ex situ* XPS spectra of AC at the selected states. The In signal in spectrum *a* came from the In film substrate to load the carbon powder for XPS tests. Due to the very few amount of carbon powder collected from the electrode, the electron beam has penetrated the carbon and reached the In substrate underneath

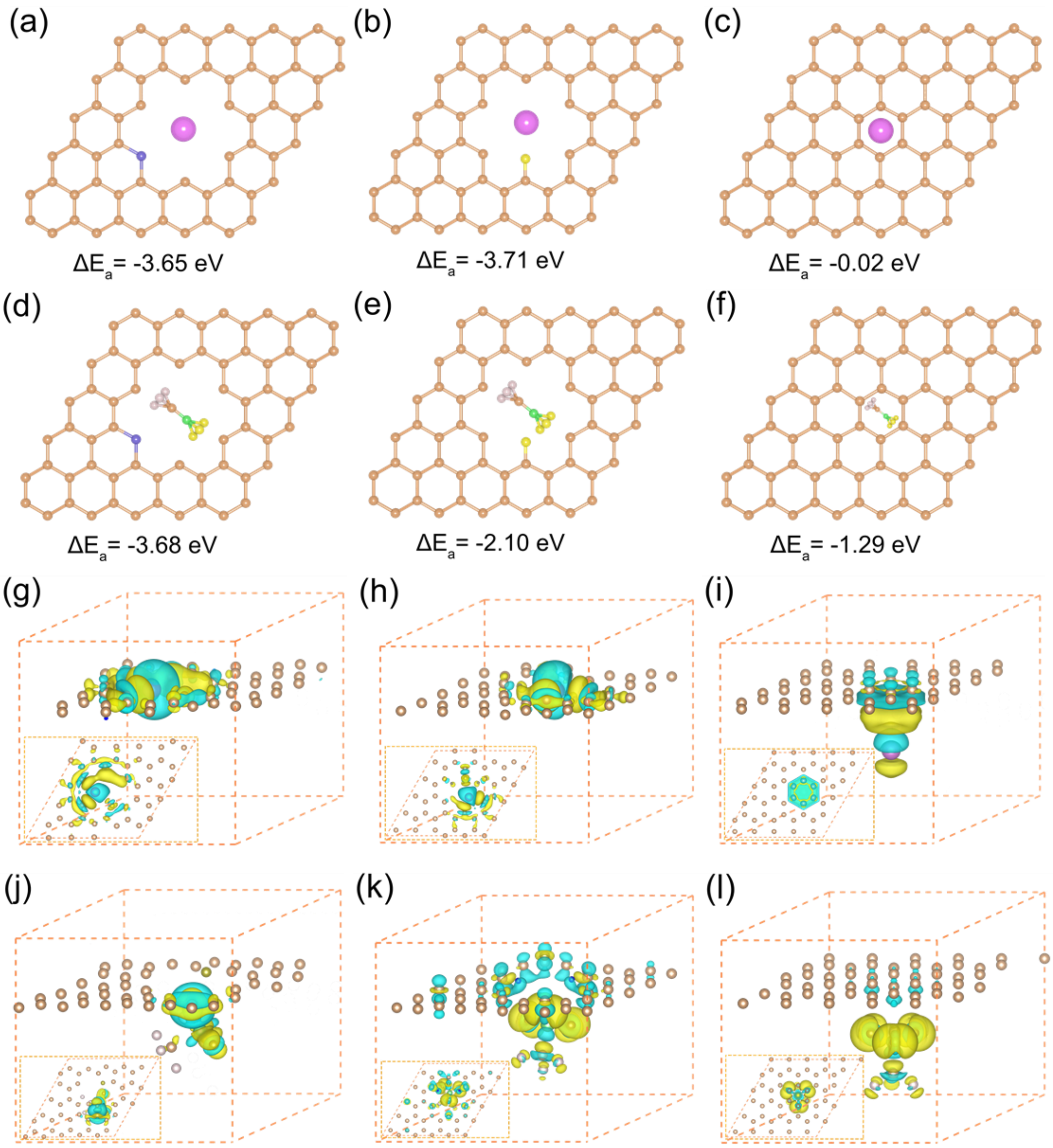

Fig. S18 Theoretical simulations of $Zn^{2+}/CF_3SO_3^-$-adsorption on different graphitic structures. The configurations and corresponding adsorption energy values of single $Zn^{2+}/CF_3SO_3^-$ adsorbed in **a**/**d** N-6, **b**/**e** O-I doped and **c**/**f** flawless graphene surface. Side and top views (inserts) of electron density differences of $Zn^{2+}/CF_3SO_3^-$ absorbed in the **g**/**j** N-6, **h**/**k** O-I doped and **i**/**l** flawless carbon structures. Yellow and blue areas represent the increased and decreased electron density, respectively. Brown, purple, light yellow, green, gray, pink and blue balls represent C, N, O, S, F, Zn and H atoms, respectively. The iso-surfaces are the 0.002 electron bohr^3

Table S5 The corresponding adsorption energy values (eV) of single Zn^{2+}/$CF_3SO_3^-$ adsorbed at on different graphitic structures

	N-6	N-5	O-I	O-II	Divacancy	Flawless
Zn^{2+}	-3.65	-4.81	-3.71	-4.58	-2.45	-0.02
$CF_3SO_3^-$	-3.68	-3.75	-2.10	-4.59	-3.53	-1.29

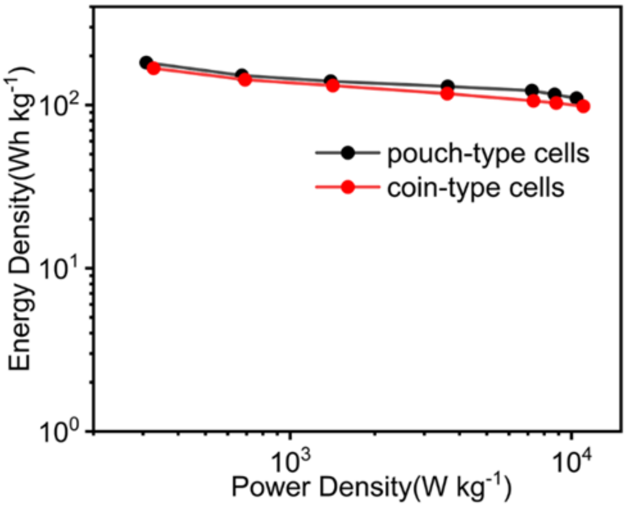

Fig. S19 Ragone plots of BGC-750 based coin cells and pouch-type cells

References

[S1]Y. Zheng, W. Zhao, D. Jia, Y. Liu, L. Cui et al., Porous carbon prepared via combustion and acid treatment as flexible zinc-ion capacitor electrode material. Chem. Eng. J. **387**, 124161 (2020). http://doi.org/10.1016/j.cej.2020.124161

[S2]S. Wu, Y. Chen, T. Jiao, J. Zhou, J. Cheng et al., An aqueous Zn-ion hybrid supercapacitor with high energy density and ultrastability up to 80000 cycles. Adv. Energy Mater. **9**, 1902915 (2019). http://doi.org/10.1002/aenm.201902915

[S3]Y. Lu, Z. Li, Z. Bai, H. Mi, C. Ji et al., High energy-power Zn-ion hybrid supercapacitors enabled by layered B/N co-doped carbon cathode. Nano Energy. **66**, 104132 (2019). http://doi.org/10.1016/j.nanoen.2019.104132

[S4]H. Zhang, Q. Liu, Y. Fang, C. Teng, X. Liu et al., Boosting Zn-ion energy storage capability of hierarchically porous carbon by promoting chemical adsorption. Adv. Mater. **31**, 1904948 (2019). http://doi.org/10.1002/adma.201904948

[S5]X. Zhang, Z. Pei, C. Wang, Z. Yuan, L. Wei et al., Flexible zinc-ion hybrid fiber capacitors with ultrahigh energy density and long cycling life for wearable electronics. Small. **15**, 1903817 (2019). http://doi.org/10.1002/smll.201903817

[S6]P. Yu, Y. Zeng, Y. Zeng, H. Dong, H. Hu et al., Achieving high-energy-density and ultra-stable zinc-ion hybrid supercapacitors by engineering hierarchical porous carbon architecture. Electrochim. Acta. **327**, 134999 (2019).

http://doi.org/10.1016/j.electacta.2019.134999

[S7]S. Chen, L. Ma, K. Zhang, M. Kamruzzaman, C. Zhi et al., A flexible solid-state zinc ion hybrid supercapacitor based on co-polymer derived hollow carbon spheres. J. Mater. Chem. A. **7**, 7784-7790 (2019). http://doi.org/10.1039/C9TA00733D

[S8]X. Li, X. Xie, R. Lv, B. Na, B. Wang et al., Nanostructured polypyrrole composite aerogels for a rechargeable flexible aqueous Zn-ion battery with high rate capabilities. Energy Technol. **7**, 1801092 (2019). http://doi.org/10.1002/ente.201801092

[S9]J. Han, K. Wang, W. Liu, C. Li, X. Sun et al., Rational design of nano-architecture composite hydrogel electrode towards high performance Zn-ion hybrid cell. Nanoscale. **10**, 13083-13091 (2018). http://doi.org/10.1039/C8NR03889A

[S10] L. Dong, X. Ma, Y. Li, L. Zhao, W. Liu et al., Extremely safe, high-rate and ultralong-life zinc-ion hybrid supercapacitors. Energy Storage Mater. **13**, 96-102 (2018). http://doi.org/10.1016/j.ensm.2018.01.003
